# Genome-wide inference reveals that feedback regulations constrain promoter-dependent transcriptional burst kinetics

**DOI:** 10.1093/nar/gkac1204

**Published:** 2022-12-30

**Authors:** Songhao Luo, Zihao Wang, Zhenquan Zhang, Tianshou Zhou, Jiajun Zhang

**Affiliations:** Guangdong Province Key Laboratory of Computational Science, Sun Yat-sen University, Guangzhou, 510275, P. R. China; School of Mathematics, Sun Yat-sen University, Guangzhou, Guangdong Province, 510275, P. R. China; Guangdong Province Key Laboratory of Computational Science, Sun Yat-sen University, Guangzhou, 510275, P. R. China; School of Mathematics, Sun Yat-sen University, Guangzhou, Guangdong Province, 510275, P. R. China; Guangdong Province Key Laboratory of Computational Science, Sun Yat-sen University, Guangzhou, 510275, P. R. China; School of Mathematics, Sun Yat-sen University, Guangzhou, Guangdong Province, 510275, P. R. China; Guangdong Province Key Laboratory of Computational Science, Sun Yat-sen University, Guangzhou, 510275, P. R. China; School of Mathematics, Sun Yat-sen University, Guangzhou, Guangdong Province, 510275, P. R. China; Guangdong Province Key Laboratory of Computational Science, Sun Yat-sen University, Guangzhou, 510275, P. R. China; School of Mathematics, Sun Yat-sen University, Guangzhou, Guangdong Province, 510275, P. R. China

## Abstract

Gene expression in mammalian cells is highly variable and episodic, resulting in a series of discontinuous bursts of mRNAs. A challenge is to understand how static promoter architecture and dynamic feedback regulations dictate bursting on a genome-wide scale. Although single-cell RNA sequencing (scRNA-seq) provides an opportunity to address this challenge, effective analytical methods are scarce. We developed an interpretable and scalable inference framework, which combined experimental data with a mechanistic model to infer transcriptional burst kinetics (sizes and frequencies) and feedback regulations. Applying this framework to scRNA-seq data generated from embryonic mouse fibroblast cells, we found Simpson's paradoxes, i.e. genome-wide burst kinetics exhibit different characteristics in two cases without and with distinguishing feedback regulations. We also showed that feedbacks differently modulate burst frequencies and sizes and conceal the effects of transcription start site distributions on burst kinetics. Notably, only in the presence of positive feedback, TATA genes are expressed with high burst frequencies and enhancer–promoter interactions mainly modulate burst frequencies. The developed inference method provided a flexible and efficient way to investigate transcriptional burst kinetics and the obtained results would be helpful for understanding cell development and fate decision.

## INTRODUCTION

The gene-expression variability resulting from programmed and stochastic processes has emerged as a central preoccupation for investigating gene regulation (1,2). Genes are stochastically transcribed often in a discontinuous bursting manner (3,4). Transcriptional bursting is regarded as a primary proxy of stochasticity in gene expression and contributes to cell-to-cell variability (5–7), but the molecular mechanisms governing transcriptional bursting kinetics still remain elusive. Many experimental studies have provided evidence for linking static promoter architecture and sequence to transcriptional bursting and, therefore, to the resulting variability in gene expression (5,8). This variability can propagate from mRNA to protein and further to the downstream target genes via a complex regulatory network (9,10). This raises important issues: On the genome-wide scale, how do static promoter regulatory sequences encode transcriptional burst kinetics, and how do dynamic gene regulatory networks shape burst kinetics?

An intuitive view is that there is an indispensable link of gene-expression variability to promoter architecture (11,12). This link is due to the fact that a basic step of RNA synthesis is to copy the genetic information from the gene promoter. Much effort has been devoted to rationalizing the promoter-architecture encoding of transcriptional burst kinetics on genome-wide scales. For example, genes with TATA boxes increase variability in expression levels, whereas the presence of CpG island significantly lowers the variability (13–15). The sharp distributions of transcription start sites (TSS) lead to higher gene-expression variability than the broad TSS distributions (13). A recent study (16) has revealed that the increases in burst sizes are dependent on the presence of TATA box and initiator elements (characteristics of the core promoter), and burst frequencies are regulated by enhancer–promoter (E–P) interactions. All these studies and others (17–21) indicate the importance of promoter architecture in modulating transcriptional burst kinetics.

Another viewpoint is that feedback regulations modulate transcriptional burst kinetics by creating a higher-level structure regulatory pattern (10). In fact, feedback regulations exist extensively in biological systems, and their functions may be reflected by the circuits of interacting genes and proteins (22). In particular, auto-regulatory feedback loops have been identified in various regulatory systems, where transcription factors directly or indirectly regulate the corresponding gene expression (23). In general, feedbacks can be categorized into positive and negative ones. Experimental investigations for a few genes or transcripts have demonstrated that different kinds of feedback played diverse roles (10). For example, negative feedback limits large expression variability and accelerates responses (24–26). Conversely, positive feedback amplifies expression variation, induces bimodal expression, and stimulates genes to ‘active’ states (27–30). In addition, negative feedback with a long delay loop can display increased variability (31). Theoretical analysis has also shown that different feedback mechanisms modulate burst kinetics in different manners (32,33). All these studies indicate the important roles of feedback regulations in mediating gene expression, including transcriptional bursts, but it is unclear whether the results obtained for case-by-case studies can hold on a genome-wide scale.

The above two viewpoints are not solitary but are complementary to each other. A challenging task is to investigate how static promoter architecture and dynamic feedback regulation coordinate transcriptional burst kinetics on a genome-wide scale. Previous studies of transcriptional bursting were limited to low-throughput experimental approaches, where observed experimental results could not be generalized across different genes or cell types (34–40). Recently, single-cell RNA sequencing (scRNA-seq) has enabled the in-depth measurement of expression levels within cell populations, providing an opportunity to study genome-wide transcriptional mechanisms (41). An important step toward this study is to develop mathematical models for the genome-wide inference of burst kinetics. The models for inference should satisfy some requirements. First, these models should be interpretable, i.e. they can capture essential gene-expression dynamics and convey kinetic information about transcriptional bursts (16,42–44) (https://doi.org/10.1101/2021.09.06.459173). Previous studies relied on inferring the direct correlations between features across molecular scales (13,45). However, these correlations are insufficient to uncover the mechanisms of gene expression. Second, the inference models should be tractable, i.e. they can effectively treat a large number of cells and genes. In general, a complex mechanistic model incorporating regulatory factors is difficult to analyze on the one hand (46), and a genome-wide inference needs expensive computational cost on the other hand. Therefore, an interpretable and tractable inference framework integrating experimental data and molecular mechanisms is strongly demanded.

Here we developed a statistical framework based on the model-driven and data-driven combination to perform a scalable genome-wide inference. This framework used the static snapshots of scRNA-seq data to infer the regulatory mechanisms underlying transcriptional burst kinetics. Specifically, it integrated the expected information on gene-expression variability, burst frequencies, burst sizes, and feedback regulation forms. Applying this inference method to the scRNA-seq data generated from embryonic mouse fibroblast cells (16), we showed that feedbacks differently modulate burst frequencies and sizes, TATA genes are expressed with high burst frequencies only in the presence of positive feedback, feedback regulations conceal the effects of TSS distribution on transcriptional burst kinetics, and E–P interactions mainly modulate burst frequencies only in the presence of positive feedbacks. Briefly, we found that characteristics of genome-wide transcriptional burst kinetics in the case without feedback regulations were different from those in the case with feedback regulations, implying Simpson's Paradox, an interesting statistical phenomenon.

## MATERIALS AND METHODS

### A mechanistic hierarchic model for statistical inference

**Figure 1.**
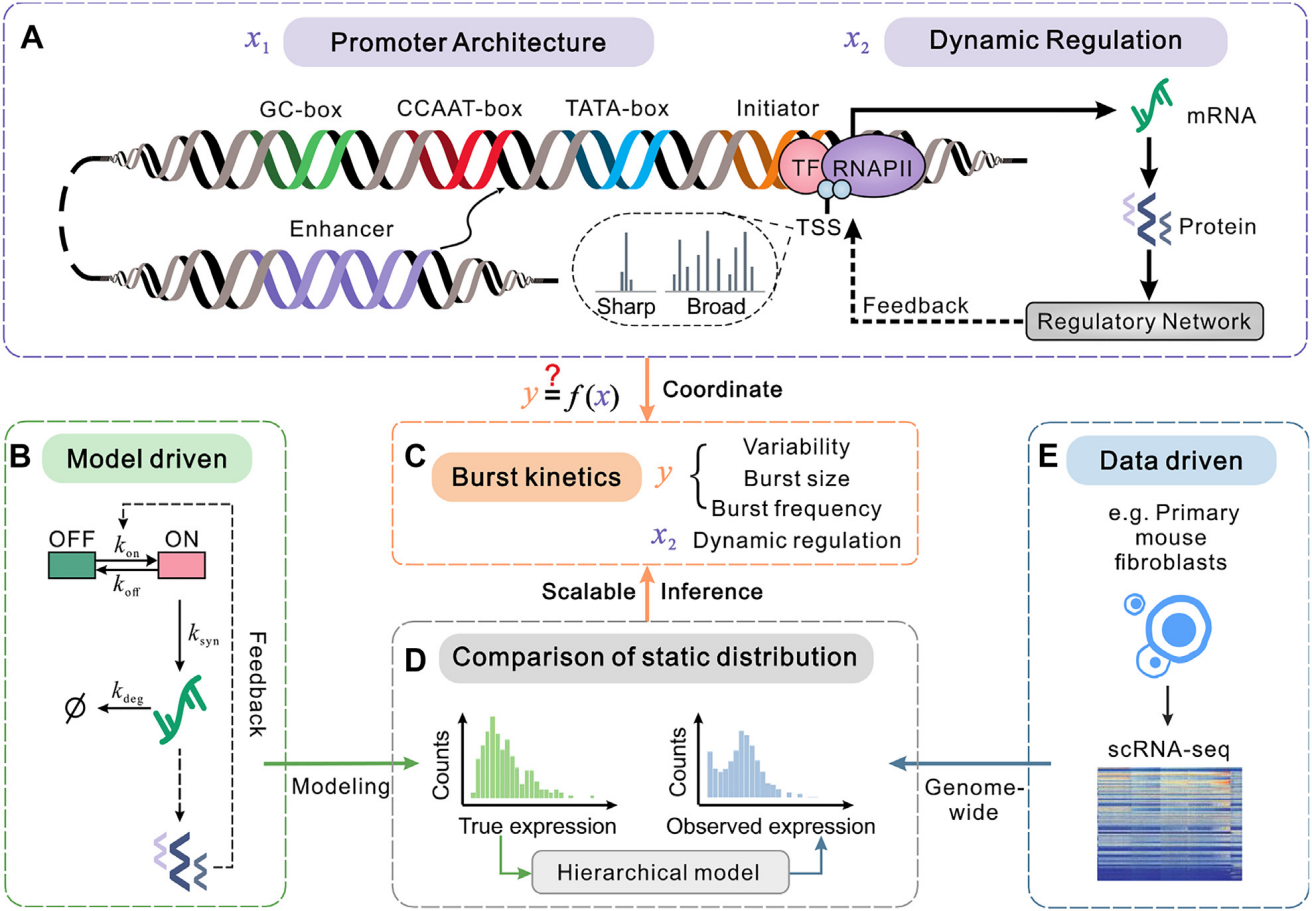
Overview of a scalable genome-wide inference method. (**A**) Schematic for important ingredients in gene expression process, including static promoter architecture information and dynamic regulation. Promoter architecture (represented by ${{\boldsymbol{x}}_1}$) consists of promoter motifs (Initiator, TATA-box, CCAAT-box, and GC-box), TSS distributions (‘sharp’ and ‘broad’) and enhancer–promoter interactions. Dynamic regulation (represented by ${{\boldsymbol{x}}_2}$) is referred to as a series of processes, such as transcription and translation as well as feedback loops, in which the gene product (as a transcription factor) regulates its own expression, possibly via a complex regulatory network. (**B**) Model-driven: schematic for a mechanistic model of stochastic gene expression, which considers an active (ON) state and an inactive (OFF) state of the promoter and auto-regulatory feedback. Here ${k_{{\rm{on}}}}$ is the switching rate from OFF to ON and ${k_{{\rm{off}}}}$ from ON to OFF; ${k_{{\rm{syn}}}}$ is the transcription rate when the gene is in ON state and ${k_{\deg }}$ is the degradation rate of mRNAs. (**C**) Kinetic parameters to be inferred, which include expression variability, burst frequency, burst size, and dynamic regulation. (**D**) Comparison between two static distributions (the left panel is for ‘true’ mRNA levels in the mechanistic gene model and the right panel for ‘observed’ mRNA counts in a given set of scRNA-seq data) by a hierarchical model can determine the values of the kinetic parameters in (C) via a scalable genome-wide inference method. (**E**) Data-driven: genome-wide scRNA-seq data of mouse embryonic fibroblasts gives an expression matrix that further gives the observed static distribution in (D).

The observed counts in a scRNA-seq experiment reflect a combination of the true expression level and the measurement level of each gene in each cell. We describe the observed counts by a two-level hierarchical model (See details in Supplementary Text 1.1, Figure 1D, and Supplementary Figure S1a–c):


(1) \begin{eqnarray*}P\left( {Y = y} \right) = \int_{0}^{\infty }{{{P_{{\rm{meas}}}}\left( {y\left| n \right.} \right){P_{{\rm{gene}}}}\left( n \right)}}dn,\end{eqnarray*}

where ${P_{{\rm{meas}}}}( {y| n } )$ is for a measurement model and ${P_{{\rm{gene}}}}( n )$ for a gene expression model.

The first level represents the measurement process for the observed count ${Y_{cg}}$ conditional on the true expression level ${N_{cg}}$ of gene *g* in cell *c*, with a conditional probability distribution (Supplementary Figure S1b):


(2) \begin{eqnarray*}\left. {{Y_{cg}}} \right|{N_{cg}}\sim{P_{{\rm{meas}}}}\left( {y\left| n \right.} \right).\end{eqnarray*}

The ${P_{{\rm{meas}}}}( {y| n } )$ describes all aspects of the technical noise produced in the measurement process for a given true expression level ${N_{cg}}$, and is suggested as a Binomial distribution or Poisson distribution which is supported by empirical analyses and theoretical arguments in many existing methods (47,48). By adding an extra sampling probability ${\lambda _{cg}}$ in the sequencing process, we characterize the sequencing depth and assume that intercellular molecules are independent of each other and only the proportional products are captured and sequenced using Binomial distribution


(3) \begin{eqnarray*}\left. {{Y_{cg}}} \right|{N_{cg}}\sim{\rm{Binomial}}\left( {n,{\lambda _{cg}}} \right).\end{eqnarray*}

In our calculations, we set the sampling probability ${\lambda _{cg}} = \lambda = 0.5$ without loss of generality since the setting of ${\lambda _{cg}}$ does not influence our qualitative results.

The second level is the true expression level of gene *g* across cells, which is assumed to follow a probability distribution


(4) \begin{eqnarray*}{N_{cg}}\sim{P_{{\rm{gene}}}}\left( n \right).\end{eqnarray*}

The underlying model describes the intrinsic dynamics of stochastic gene expression. How an appropriate gene-expression model ${P_{{\rm{gene}}}}( n )$ is chosen is critical. In general, this choice needs to satisfy two basic requirements: (i) the model should capture the essential gene-expression dynamics of interest (e.g. transcriptional burst kinetics); and (ii) the inference based on the model should be effective and scalable to large numbers of cells and genes. As combining mechanistic models to infer the entire gene regulatory network would lead to sophisticated models that become intractable, we simplified the gene regulatory network to a feedback loop, which is the most common form existing in gene expression systems. For these purposes, we adopt a model of stochastic gene expression (Supplementary Text 1.1, Figure 1B and Supplementary Figure S1c), which simultaneously characterizes transcriptional burst kinetics and auto-regulatory feedbacks with the below distribution


(5) $$\begin{eqnarray*} && {P_{{\rm{gene}}}}\left( n \right) = \nonumber\\ && \mathcal{N}\int_{0}^{\infty }{{{\rm{Poisson}}\left( {n\left| x \right.} \right){x^{a\left( {1 + \varepsilon } \right) - 1}}{e^{ - {x \mathord{\left/ {\vphantom {x b}} \right. } b}}}{{\left( {1 + {{\left( {{x \mathord{\left/ {\vphantom {x k}} \right. } k}} \right)}^h}} \right)}^{{{ - a} \mathord{\left/ {\vphantom {{ - a} h}} \right. } h}}}dx}}, \end{eqnarray*}$$

where $\mathcal{N}$ is a normalization factor. Note that the discrete gene expression distribution (Equation (5)) is a Poisson representation in form, i.e. ${P_{{\rm{gene}}}}( n ) = \int_{0}^{\infty }{{{\rm{Poisson}}( {n| x } )f( x )dx}}$, where $f( x )$ is a kernel density function that has the same form as the continuous distribution of proteins in (49). As illustrated in Figure 1B, this kernel function $f( x )$ can extract several kinetic parameters, denoted by $\theta = ( {a,b,\varepsilon ,k,h} )$, of the steady-state gene-product distribution from a dynamic model with auto-regulatory feedback described by some meaningful kinetic parameters: switching rates between inactive and active state (${k_{{\rm{on}}}}$, ${k_{{\rm{off}}}}$), mRNA transcription rates (${k_{{\rm{syn}}}}$) and degradation rates (${k_{{\rm{deg}}}}$). Here, $a$ is the number of bursts per cell cycle (burst frequency), and $b$ is the mean number of gene products generated per burst (burst size), and $h$ is a vital parameter of capturing the feedback regulation dynamics of gene products, which is actually a Hill coefficient. Furthermore, this model can describe two most common feedback loops in gene expression: positive-feedback loop (i.e. $h < 0$) and negative feedback loop (i.e. $h >0$). It should be noted that the auto-regulatory feedbacks involve gene products, which directly or indirectly regulate the corresponding target gene itself through feedback loops, resulting in a repressing or activating expression. The small leakiness proportion of the promoter $\varepsilon$ contains the information on the baseline bursts in the absence of regulation, and $k$ contains the information on the equilibrium binding constant (see details in Supplementary Text 1.1). Overall, Equation (5) is a mechanistic model, which can simultaneously describe the burst-production and feedback-regulation processes of gene expression. More importantly, as a special case of this model, $h = 0$ corresponds to the negative binomial distribution of gene expression ${P_{{\rm{gene}}}}( n )$ (i.e. non-feedback).

By combining Equations (3) and (5) and substituting into Equation (1), the discrete probability distribution of ${Y_{cg}}$ can be computed but is expressed in an integral form (see details in Supplementary Text 1.1):


(6) $$\begin{eqnarray*} &&P\left( {Y = y; \cdot } \right) = \int_{0}^{\infty }{{{P_{{\rm{meas}}}}\left( {y\left| n \right.} \right){P_{{\rm{gene}}}}\left( n \right)}}dn\nonumber\\ &&= \int_{0}^{\infty }{{{\rm{Poisson}}\left( {Y = y\left| {\lambda x} \right.} \right)}}f\left( x \right)dx\nonumber\\ &&= \mathcal{N} \cdot {\rm{ }}\int_{0}^{\infty }{{\frac{{{{\left( {\lambda x} \right)}^y}}}{{y!}}}}{e^{ - \lambda x}}{x^{a\left( {1 + \varepsilon } \right) - 1}}{e^{ - x/b}}{\left( {1 + {{\left( {x/k} \right)}^h}} \right)^{ - a/h}}dx. \end{eqnarray*}$$

For the case of non-feedback, we employ the negative binomial distribution, which is then given by


(7) \begin{eqnarray*}P\left( {Y = y; \cdot } \right) = \int_{0}^{\infty }{{\frac{{{{\left( {\lambda x} \right)}^y}}}{{y!}}}}{e^{ - \lambda x}}\frac{1}{{{b^a}\Gamma \left( a \right)}}{x^{a - 1}}{e^{ - x/b}}dx.\end{eqnarray*}

where $\Gamma ( \cdot )$ is the Gamma function.

### Maximum likelihood estimation of parameters

Here we introduce a method of estimating the kinetic parameters in our hierarchical model using the expression data of each gene. For a given expression read of observed cells, the most common parameter estimation method is the maximum likelihood estimation, which can be formulated as the following optimization problem of five parameters $\theta = ( {a,b,\varepsilon ,k,h} )$ in our case


(8) \begin{eqnarray*}\mathop {\arg }\limits_\theta \min \left( { - L\left( {y;\theta } \right)} \right) = \mathop {\arg }\limits_\theta \min \sum\limits_y { - \ln (P(Y = y;\theta ))} ,\end{eqnarray*}

where $P(Y = y;\theta )$ is described in Equation (6).

Because of the complex integral and unnormalized probability mass function in Equation (6), calculating the integral directly through the MCMC method (50) would be at a high cost of computation, and in particular, it is hard to use in the analysis of genome-wide data. Therefore, we apply the Generalized Gauss-Laguerre Quadrature Rules (51) to Equation (6) instead of the use of the MCMC method, realizing a rapid calculation in inference:


(9) \begin{eqnarray*} &&P\left( {Y = y;\theta } \right){\rm{ = }}\mathcal{N} \int_{0}^{\infty }{{\frac{{{{\left( {\lambda x} \right)}^y}}}{{y!}}}}{e^{ - \lambda x}}{x^{a\left( {1 + \varepsilon } \right) - 1}}{e^{ - x/b}}{\left( {1 + {{\left( {x/k} \right)}^h}} \right)^{ - a/h}}dx\nonumber\\ && \approx \mathcal{N} \sum\limits_{i = 1}^n {{w_i}f\left( {{x_i}} \right)} , \end{eqnarray*}

where ${x_i},{\rm{ }}{w_i}$ can be determined by the generalized Laguerre polynomials.

In particular, a simple algebraic transformation in Equation (7) yields the explicit expression of the probability distribution in the case of non-feedback:


(10) \begin{eqnarray*}&& P\left( {Y = y; \cdot } \right) = \int_{0}^{\infty }{{\frac{{{{\left( {\lambda x} \right)}^y}}}{{y!}}}}{e^{ - \lambda x}}\frac{1}{{{b^a}\Gamma \left( a \right)}}{x^{a - 1}}{e^{ - x/b}}dx\nonumber\\ &&= \frac{{{{(b\lambda )}^y}}}{{\left( {a + y} \right)B\left( {a,y + 1} \right){{\left( {b\lambda + 1} \right)}^{y + a}}}}, \end{eqnarray*}

where $B( { \cdot , \cdot } )$ is the Beta function.

### Optimization method and initial values setting

To realize a fast calculation for solving the optimization problem (Equation (8)) of parameter estimation, we use the *fmincon* function in the LBFGS method of MATLAB (https://www.mathworks.com/products/matlab.html), a nonlinear programming solver, to find the minimum of the optimization problem given a set of initial values and parameter intervals $a = ( {{{10}^{ - 1}},30} ),{\rm{ }}b = ( {{\rm{1}},20} ),{\rm{ }}k = ( {{\rm{1}},{\rm{1}}{{\rm{0}}^3}} ),{\rm{ }}h = ( { - {\rm{10}}, - {\rm{1}}} ){\rm{ or }}( {1,{\rm{10}}} )$. For each gene and each case of positive, negative, and non-feedbacks, we repeatedly solve the optimization problem 30 times.

We restrict that the initial values of the optimization problem obey the following rules. First, we consider initial points of $a$ (burst frequency) and $b$ (burst size). Here $X$ is a random variable of the distribution in Equation (5). Since Gamma distribution is a special case of Equation (5), we assume that the initial points $a$ and $b$ follow


(11) \begin{eqnarray*}{\rm{E}}\left[ X \right] = ab,{\rm{ Var}}\left[ X \right] = a{b^2}.\end{eqnarray*}

On the other hand, by considering the initial values of $Y$ generated by our hierarchical model, we have


(12) \begin{eqnarray*}{\rm{E}}\left[ Y \right] = {\rm{E}}\left[ {{\rm{E}}\left[ {Y\left| X \right.} \right]} \right]{\rm{ = }}\lambda {\rm{E}}\left[ X \right] = \lambda ab,\end{eqnarray*}

(13) \begin{eqnarray*}{\rm{Var}}\left[ Y \right] = {\rm{E}}\left[ {{\rm{Var}}\left[ {Y\left| X \right.} \right]} \right] + {\rm{Var}}\left[ {{\rm{E}}\left[ {Y\left| X \right.} \right]} \right] = \lambda ab + {\lambda ^2}a{b^2}.\end{eqnarray*}

Given the expectation and variance of $Y$, we can in return estimate burst frequency $a$ and burst size $b$ by rearranging Equations (12) and (13), which are then taken as initial values


(14) $$\begin{equation*}a = \frac{{{\rm{E}}{{\left[ Y \right]}^2}}}{{{\rm{Var}}\left[ Y \right] - {\rm{E}}\left[ Y \right]}},{\rm{ }}b = {{\left( {\frac{{{\rm{Var}}\left[ Y \right]}}{{{\rm{E}}\left[ Y \right]}} - 1} \right)} \mathord{\left/ {\vphantom {{\left( {\frac{{{\rm{Var}}\left[ Y \right]}}{{{\rm{E}}\left[ Y \right]}} - 1} \right)} \lambda }} \right. } \lambda }.\end{equation*}$$

Usually, the transcriptional rate in the OFF state is much smaller than that in the ON state. And a small enough leaky rate does not affect the distribution shape of gene expression. Therefore, we fix $\varepsilon$ at the constant of 0.05 during our inference. The $h$ value is extracted from a uniform distribution between the integer -5 and -1 (positive feedback) or integers 1 and 5 (negative feedback). And the values of parameter $k$ are extracted from the log-uniform (logarithm base 10) distribution on interval $[ {0,2} ]$.

### Model selection

Using the above inference method, we obtain 90 inferred results for each case of positive, negative, and non-feedbacks. Then, we filter out the unreliable results on the inference boundary, which are possibly caused by the optimization program setting. On this basis, we compute the value of the corrected Akaike information criterion (AICc) (52) and select the best model corresponding to the smallest AICc,


(15) \begin{eqnarray*}{\rm{AICc}} = - 2\log L\left( {\hat{\theta }} \right) + 2k + \frac{{2k\left( {k + 1} \right)}}{{n - k - 1}},\end{eqnarray*}

where the maximum likelihood $L( {\hat{\theta }} )$ is the result during the inference run, $k$ is the number of model parameters, and $n$ is the sample size of observed data.

### Validation on synthetic scRNA-seq data

In order to check whether the above statistical inference method can effectively infer burst frequency, burst size, and feedback form in our hierarchical model, we produce synthetic single-cell RNA data. Given a set of model parameter values $\theta = ( {a,b,\varepsilon ,k,h} )$, we first calculate the probability distributions of these parameters according to the method described in the above section and then carry out random samples according to the probability of each parameter value to obtain the input data for the inference process.

We show the precision regions for the inference of burst kinetics (burst frequencies and burst sizes) under different feedback strengths $h$ and different equilibrium binding constants $k$ (Supplementary Figure S4–S6). And the error between true parameters ${\theta ^{{\rm{true}}}} = ( {b{f^{{\rm{true}}}},b{s^{{\rm{true}}}}} )$ and estimated parameters ${\theta ^{{\rm{est}}}} = ( {b{f^{{\rm{est}}}},b{s^{{\rm{est}}}}} )$ is calculated according to:


(16) \begin{eqnarray*}{\rm{Error}}\left( {{\theta ^{{\rm{true}}}},{\theta ^{{\rm{est}}}}} \right) & = & {\left( {\log \left( {b{f^{{\rm{true}}}}} \right) - \log \left( {b{f^{{\rm{est}}}}} \right)} \right)^2}\nonumber\\ && + {\left( {\log \left( {b{s^{{\rm{true}}}}} \right) - \log \left( {b{s^{{\rm{est}}}}} \right)} \right)^2}.\end{eqnarray*}

We show the robustness of the inference in the cases of positive, negative, and non-feedbacks, respectively (Supplementary Figure S4–S6). To explore the robustness of the cell numbers to the inference, we select different sampled cell numbers (200, 300, 500, 1000, 5000) to synthesize data 50 times, and at each time, set 30 different initial points for optimization in each case of feedback forms. The optimization process is the same as the inference process of real data. The same process is used to explore the effects of stochastic losses of mRNA molecules (sensitivity), missing randomly at a certain probability (0.1, 0.3, 0.5, 0.7 or 0.9) from sufficient samples (number = 2000). The results of inference robustness analysis are illustrated with two different distribution examples in the three cases of feedback forms (Supplementary Figure S4–S6).

### Inference evaluation

To assess whether the observed data came from the distribution generated via the parameters inferred by our method, we use goodness-of-fit statistics that obey chi-square distribution of large samples:


(17) \begin{eqnarray*}{\chi ^{\rm{2}}} = \sum\limits_{k = 0}^\infty {\frac{{{{\left( {{O_k} - {E_k}} \right)}^2}}}{{{E_k}}}} ,\end{eqnarray*}

where ${O_k}$ is the observed sample number whose mRNA number is *k*, and ${E_k}$ is the expected sample number. Note that in some sequencing techniques, the cell samples of scRNA-seq data are not large enough, so it is needed to use the Monte Carlo method to generate the null distribution of chi-square goodness-of-fit test instead of the asymptotic distribution. For each gene, we first generate the same number of samples as that in the observed data from the probability of each point with the inferred parameters and then compute the $\chi _{sim}^{\rm{2}}$ statistic according to Equation (17). After repeating the Monte Carlo simulation procedure for 1000 times, we judge whether the resulting inference is a good fit by comparing $\chi _{obs}^2$ with the resulting 1000 $\chi _{sim}^{\rm{2}}$. The criterion that an inferred parameter is a good fit is that the $\chi _{obs}^2$ is at least less than five percentage numbers of $\chi _{sim}^{\rm{2}}$ (Supplementary Figure S7a).

### Data analysis

#### scRNA-seq data processing

We utilize the processed scRNA-seq data for 10727 genes of transcriptomes from 224 individual mouse embryonic fibroblasts for each allele (C57 × CAST) (16). In that paper, the quantification of gene transcription is based on the Smart-seq2 scRNA-seq libraries, and UMI counts is used to reduce the amplification noise. To ensure that the inference process is not hindered by low-quality elements of the data as far as possible, we carry out a certain degree of quality control of the original data (from the file: SS3_cast_UMIs_concat.csv and SS3_c57_UMIs_concat.csv). We filter out the genes expressed in less than 50 cells. Also, we filter out the cells expressed in <2000 genes. In addition, we filter out the genes whose overall average expression levels are <2. After these manipulations on each allelic data (C57 × CAST), the genes that meet the conditions are combined to facilitate inferences from more adequate samples and give a single-cell expression matrix composed of 2162 genes and 413 cells. This treatment is based on the assumption that the distributions of almost all genes for the CAST and c57 alleles have similar shapes and that the transcriptional dynamic behavior is consistent between alleles for most genes, which is also supported by previous studies (16,53). And, we removed the outlier data with the tail 5% of the distribution. In addition, our method can be also applied to any high-quality non-allelic scRNA-seq data.

#### Identification of promoter motif and TSS distribution

The recognition and coordinates of the promoter motifs (TATA box, Initiator, CCAAT box, GC box) are downloaded from ‘the Select/Download Tool’ of the EPD New database (54). In order to determine the TSS distribution of mouse embryonic fibroblasts, MEFs FANTOM5 Cap Analysis of gene expression data is retrieved through the CAGEr R package (55). After normalization and TSS clustering, TSS distribution is defined as ‘sharp’ if the promoter width is less than 15bp (this length is taken as the median of all genes), and as ‘broad’ otherwise.

#### Identification of enhancer–promoter intensity

The data about the interaction between enhancer and promoter is downloaded from (16). The dataset is used to compare the correlation between burst kinetics and enhancer activity of fibroblasts and mESCs. Enhancer activity is calculated according to the intensity of the H3K27ac peak measured in the defined EPUs region (which is considered that enhancer and promoter interactions occur more possibly) via ChIP-seq in a previous study (56). In our study, we only utilize the collated data that includes the peak of H3K27ac in EPUs of MEFs.

### Statistical analysis

#### Gene expression variability

Gene expression variability is usually quantified by the square of the coefficient of variation (CV^2^), which is defined as the ratio of the variance over the square of the mean. According to this definition, we calculate gene-expression variability in a given set of observed data $Y$ for gene $g$. Similarly, we use the inferred ${\theta ^{{\rm{est}}}}$ to calculate the theoretical CV^2^ of our hierarchical model for gene $g$, that is,


(18) \begin{eqnarray*}{\rm{CV}}_g^2 = \frac{{{\rm{Var}}\left[ {Y;\theta _g^{est}} \right]}}{{{\rm{E}}{{\left[ {Y;\theta _g^{est}} \right]}^2}}}.\end{eqnarray*}

When fitting CV^2^ with a cubic spline, we find that there is a strong correlation between the mean expression level and CV^2^ (Supplementary Figure S9a). Many studies have discussed the relationship between gene-expression variability and mean (57,58). Note that in the classical telegraph model, the total mRNA variability can be decomposed into two parts: the mRNA internal variability generated from transcription and the promoter variability due to the switching between active and inactive states. Inspired by (15), we adjust the variability by subtracting the inverse of the logarithmic mean (logarithm base 2), thus obtaining the residual squared coefficient of variation (*r*CV^2^). For example, for gene $g$, we have


(19) \begin{eqnarray*}r{\rm{CV}}_{^g}^2 = {\rm{CV}}_g^2 - \frac{1}{{{{\log }_2}\left( {{\mu _g}} \right)}},\end{eqnarray*}

where ${\mu _g} = {\rm{E}}[ {Y;\theta _g^{est}} ]$. As a result, the influence of the mean expression level on the expression variability is basically eliminated after performing a linear regression on *r*CV^2^ (Supplementary Figure S9b).

#### Linear regression model in promoter motif analysis

After having obtained the promoter motifs of each gene from the EPD database and its burst kinetics (*r*CV^2^, burst frequency, burst size) by inference, we conduct multivariate linear regression with interaction terms to find the correlations between quantities of interest in cases of positive, negative, and non-feedbacks. Specifically, we perform multivariate linear regression according to


(20) \begin{eqnarray*}\begin{array}{@{}c@{}} r{\rm{C}}{{\rm{V}}^2} \sim \left( {TATA * Inr + CCAAT * GC} \right) \times feedback,\\ {\log _{10}}\left( {bf} \right) \sim \left( {TATA * Inr + CCAAT * GC} \right) \times feedback,\\ {\log _{10}}\left( {bs} \right) \sim \left( {TATA * Inr + CCAAT * GC} \right) \times feedback. \end{array}\end{eqnarray*}

In Figure 4B–D and Supplementary Figure S10c–e, we show the *t*-value in the regression results. The absolute of *t*-value is larger in the test of the linear regression coefficient, indicating that the resulting correlations are significant.

## RESULTS

### An integrated statistical framework for learning promoter-dependent yet feedback-constrained transcriptional burst kinetics on a genome-wide scale

Cell-to-cell heterogeneity in gene expression is primarily attributed to transcriptional bursting (12,59,60), which is represented by a vector ${\boldsymbol{y}}$ of components including burst frequency, burst size, expression variability, etc. (Figure 1C). Transcriptional bursts result from complex molecular processes on multilayered sources (1), which are represented by a vector ${\boldsymbol{x}}$ of components including static DNA sequences, epigenetic modifications (61), transcription, translation, dynamic network regulations, etc. (2). Then, the question of how these molecular processes coordinate transcriptional busting can be mathematically described as ${\boldsymbol{y}} = f( {\boldsymbol{x}} )$, where $f$ is a vector function describing the correlation of ${\boldsymbol{x}}$ to ${\boldsymbol{y}}$.

Static promoter architecture is an essential DNA sequence for binding transcription factors during mRNA synthesis. Specifically, promoter motifs (such as initiator, TATA-box, CCAAT-box, GC-box), broad and sharp TSS distributions, and enhancer–promoter interactions are essential features of eukaryotic promoter architecture (Figure 1A, left). Meanwhile, variability in gene expression can propagate from mRNA to protein and further to target genes, possibly through a dynamic and complex gene regulatory network. A common form of dynamic regulation is auto-regulation which directly or indirectly regulates the corresponding target gene itself through a feedback loop, resulting in a repressing or activating expression (Figure 1A, right). For clarity, we let vectors ${{\boldsymbol{x}}_1}$ and ${{\boldsymbol{x}}_2}$ represent static promoter architecture and dynamic feedback regulation, respectively (Figure 1A). The information on promoter architecture (${{\boldsymbol{x}}_1}$) can be recovered from public bioinformatics databases such as the EPD database (54), Bioconductor (62), and UCSC Genome Browser (63). In general, the mechanisms of dynamic regulation (${{\boldsymbol{x}}_2}$) and burst kinetics (${\boldsymbol{y}}$) are not directly measurable but hidden in data sets. Unlike some imaging-based technologies such as MS2 system (64) that were limited to a few genes and could not be extended to the whole genome, single-cell sequencing technologies made it possible to recover the information on dynamic regulations (${{\boldsymbol{x}}_2}$) and burst kinetics (${\boldsymbol{y}}$) from static snapshots (Figure 1E). Figure 1B–E summarizes the genome-wide inference procedure proposed here. This procedure used a statistical framework of the model-driven (Figure 1B) and data-driven (Figure 1E) combination to infer dynamic feedback regulations and transcriptional burst kinetics from static scRNA-seq data (Figure 1C, D) under the assumption that the abundances of mRNA and protein were highly dependent (65).

Specifically, our statistical inference framework used a mechanistic model of gene expression (Figure 1B), which simultaneously considered transcriptional burst kinetics (${\boldsymbol{y}}$) and feedback regulations (${{\boldsymbol{x}}_2}$), to obtain ‘true’ gene expression distributions (Figure 1D, left). On the other hand, the known scRNA-seq data gave ‘observed’ gene expression distributions, implying possible errors in the sequencing technologies (66,67). A hierarchical statistical model (see ‘Materials and Methods’) was proposed to link ‘true’ gene-expression levels (Figure 1D, left) and ‘observed’ mRNA counts (Figure 1D, right), thus determining key kinetic parameters (expression variability, burst size, burst frequency, and dynamic regulation) (Figure 1C). We emphasized that the proposed framework was a scalable genome-wide inference, which was particularly useful in revealing how both static promoter architecture and dynamic feedback regulation coordinate transcriptional bursting.

### A hierarchical model provides the genome-wide inference of transcriptional burst kinetics and feedback regulations from single-cell snapshots

The hierarchical statistical model developed here can give a mechanistic interpretation for Unique Molecular Identifiers (UMIs) based on scRNA-seq data. In fact, this model not only captured the characteristics of transcriptional burst kinetics and feedback regulations, but also described the measured noise of UMIs data (see ‘Materials and Methods’). Then, we used the maximum likelihood method to determine burst kinetics and feedback forms (positive-, negative-, non-feedback) within biologically reasonable ranges of model parameters. Note that the inferences with traditional MCMC methods (50) would need huge and even unaffordable computational costs since the static mRNA distribution was expressed in a high-order integral that is difficult to solve. To overcome this difficulty, we developed a fast algorithm for computing this distribution based on generalized Gauss-Laguerre quadrature rules, thus realizing a scalable genome-wide inference (51) (see ‘Materials and Methods’).

To evaluate the validity of our inference method, we first explored the sensitivity of distribution shapes to changes in model parameters. We found that the genes with high expression levels were more sensitive to model parameters than the other genes (Supplementary Figures S2 and S3). Then, to test the reliability of the method in inferring kinetic parameters, we generated synthetic single-cell RNA data by stochastic sampling from the distribution for the hierarchical model with known parameter values. Through inference using the synthetic data, we can give robust estimates of burst frequencies, burst sizes, and feedback forms from the corresponding static mRNA distributions (Supplementary Figure S4–S6). Besides, we also assessed the robustness of our inference method to different cell numbers and stochastic losses of mRNA molecules (mimicking the incomplete mRNA detection in scRNA-seq protocols) (Supplementary Figures S4b–c, S5b–c and S6b–c). Overall, we provided a mechanistic model and an effective, robust and scalable inference method for learning dynamic burst kinetics and feedback forms from static snapshot data, which can be conveniently used in the analysis of scRNA-seq data.

**Figure 2.**
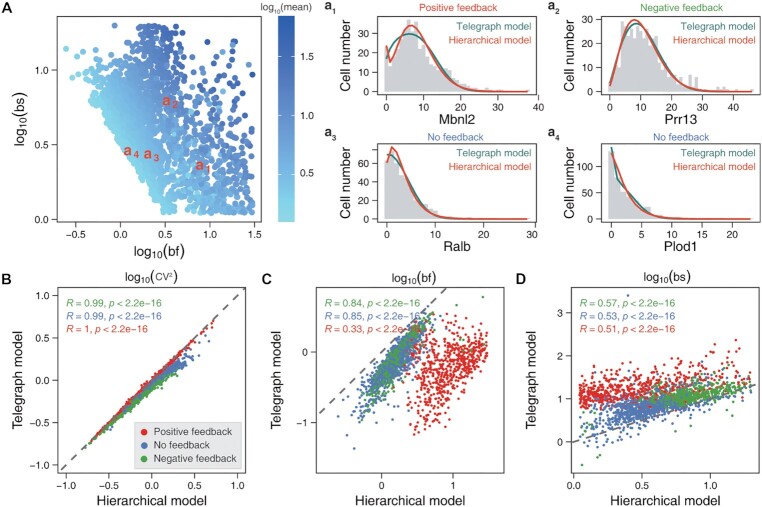
Genome-wide characteristics of transcriptional burst kinetics inferred from the scRNA-seq data of primary mouse fibroblasts. (**A**) Scatter plots of burst frequencies (bf) and burst sizes (bs), where the colored points represent mean expression levels. **a_1_–a_4_** Examples for comparison of the inferred distributions between our hierarchical model (orange line) and the telegraph model (green line), where the gray histograms represent the distributions of mRNA counts. (**B–D**) Scatter plots of the expression variability (CV^2^, B), burst frequencies (C) and burst sizes (D), which are correlated in the sense of Pearson correlation test (see the indicated values of *R* and *P*-value). The values of these kinetic parameters are obtained via the hierarchical model and the telegraph model, respectively. Red dots correspond to positive feedback, blue dots to non-feedback, and green dots to negative feedback. The slope of dashed lines equals 1.

Next, we applied our hierarchical model and inference approach to the scRNA-seq data of primary mouse fibroblasts (16). From the original UMIs data containing 10727 genes and 224 cells, we selected 2162 highly expressed genes using a quality control method and then merged two allelic expression data into a matrix to infer burst kinetics and feedback forms. We observed that these selected genes were transcribed with widely different burst kinetics (68), and in particular, those genes with the same average expression level exhibited diverse burst kinetics, implying that the expressions of different genes were regulated possibly by different molecular mechanisms (Figure 2A). To check the validity of these inferred results, we performed a goodness-of-fit test (see ‘Materials and Methods’). We found that the distributions from the dataset were consistent with those obtained using the inferred parameters (Supplementary Figure S7a), and confirmed that the mRNA mean and variability in the mechanistic model matched those in the data (Supplementary Figure S7b). All the good-fit genes can be classified into three categories: 626 positive-feedback genes, 625 negative-feedback genes, and 840 non-feedback genes. The inferred results for example genes: Mbnl2, Prr13, Ralb, and Plod1 were demonstrated in Figure 2a_1_-a_4_, showing that these genes had different feedback forms and followed different distributions. Interestingly, our hierarchical model can particularly recover bimodal distributions from static data, which however were fitted as unimodal distributions via the telegraph model without feedback (69) (e.g. the distribution of the Mbnl2 gene as shown in Figure 2a_1_ and more genes as shown in Supplementary Figure S8). In addition, we compared the inferred results between our hierarchical model and the telegraph model, finding that both models captured almost the same gene-expression variability (CV^2^, Figure 2B) while keeping high correlations between burst frequencies and burst sizes (Figure 2C, D, *P*-value < 2.2 × 10^–16^). Notably, we found that the forms of dynamic feedback regulations can lead to different burst kinetics on a genome-wide scale but cannot be inferred by previous methods (Figure 2C, D) (16,43).

### Feedbacks modulate burst frequencies and sizes differently

**Figure 3.**
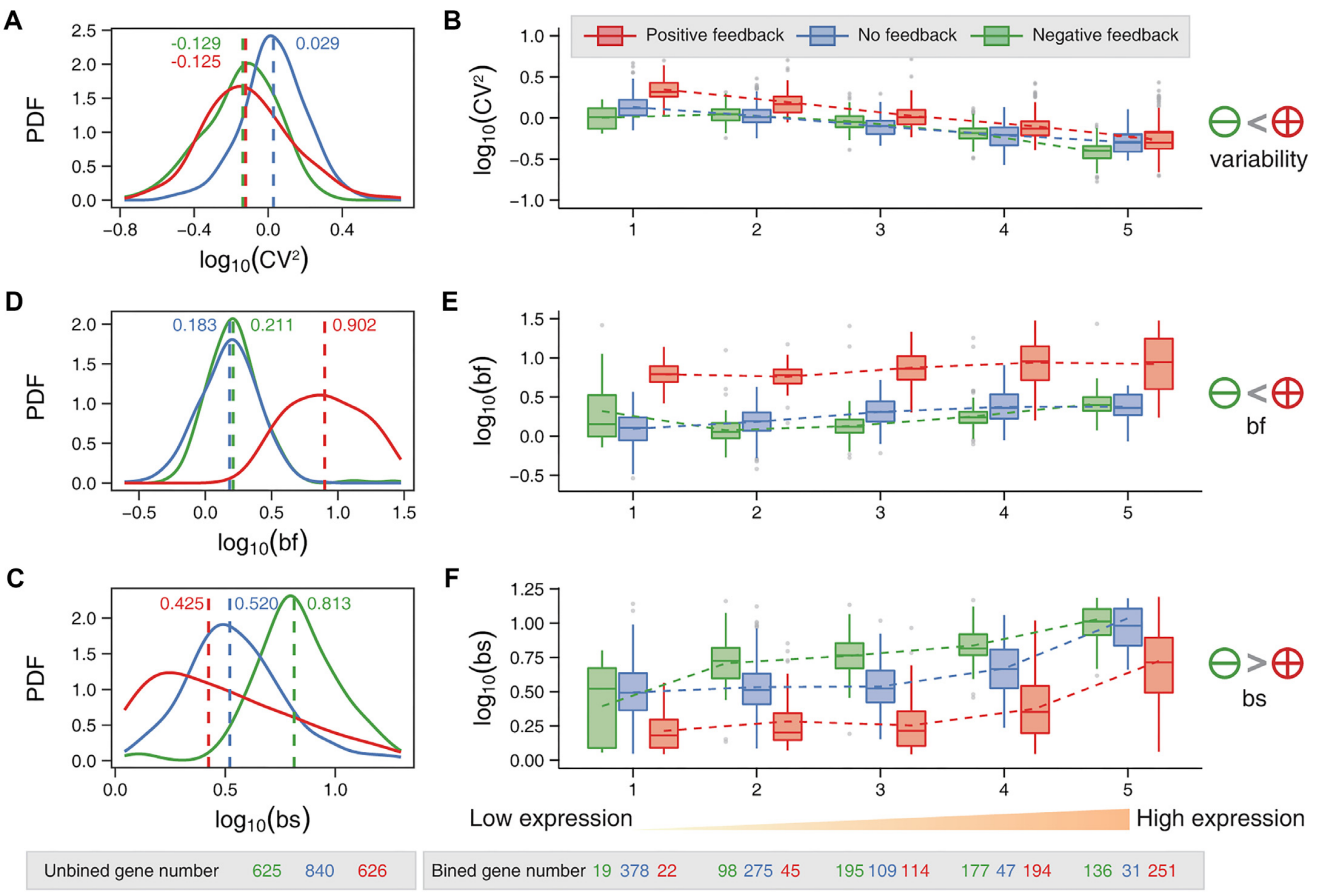
Genome-wide comparison of transcriptional burst kinetics in three cases of feedback regulation. (**A–C**). Three probability density functions (PDF) of expression variability (CV^2^, A), burst frequencies (bf, B), and burst sizes (bs, C) for positive-feedback genes (red), non-feedback genes (blue) and negative-feedback genes (green), where dashed lines represent the medians. (**D–F**) Boxplots of expression variability (D), burst frequency (E) and burst size (F). The genes are divided into five boxes with an equal number of genes, and the gene-expression level increases from left to right, where the dashed line connects the mean expression levels in each box. The number of good-fit genes per feedback type is shown at the bottom of the figure.

Having inferred each gene's burst kinetics and feedback forms, we next investigated how feedback regulations affected expression variability (CV^2^) and transcriptional burst kinetics on a genome-wide scale. Interestingly, we found the statistical phenomenon of Simpson's paradox. First, we observed from Figure 3A that there were no significant differences in variability distributions between the positive-feedback and the negative-feedback genes, but the non-feedback genes exhibited higher expression variability. The latter result seemed inconsistent with the previous conclusions that positive feedback amplified variability and negative feedback attenuated variability (70). This can be interpreted by the fact that the expression level and the expression variability were negatively correlated (57,58,71) (Supplementary Figure S9a). To show this point, we introduced the average expressed variable by dividing all the selected genes into five equal boxes based on average expression levels and tracked the expression-variability changes when the average gene-expression levels were increased. Then, we found that the expression variability was indeed negatively correlated with the average expression levels, regardless of feedback forms (Figure 3D). Furthermore, the positive-feedback genes showed relatively higher expression variability than the negative-feedback genes at the same expression levels (Figure 3D), consistent with the results obtained in previous studies (70,72).

Next, we checked the genome-wide effects of feedback regulations on transcriptional burst frequencies and burst sizes. Interestingly, we found that positive and negative feedback differently modulated burst frequencies and sizes (Figures 3B, C, and Supplementary Figures S9c, d). Specifically, the burst frequencies of positive-feedback genes were significantly higher than those of negative-feedback genes on the whole genome (Figure 3B) and at the same expression level (Figure 3E). By contrast, the burst sizes of positive-feedback genes were smaller than those of negative-feedback genes (Figure 3C, F). In addition, the effects of negative feedback and non-feedback on burst frequencies were difficult to distinguish (Figure 3B, E), but there was a significant difference in burst sizes (Figure 3C, F). This observation suggested that burst size could be a distinguishable characteristic between negative-feedback and non-feedback genes.

Finally, in this subsection, we point out that an unexplored issue is how promoter architecture affects transcriptional burst kinetics in the presence of feedback regulation on a genome-wide scale. Below, we address this issue from three aspects: promoter motifs, TSS distributions, and enhancer–promoter interactions in the following.

### TATA genes are expressed with high burst frequencies only in the presence of positive feedback

It was reported that promoter motifs such as TATA box and initiator regulated transcriptional bursting directly (13,14,16,57,73). On the other hand, we showed in the previous section that different feedback regulations led to different burst kinetics. This raised an unexplored question: how do promoter motifs modulate transcriptional burst kinetics in the presence of feedback regulation on the genome-wide scale?

**Figure 4.**
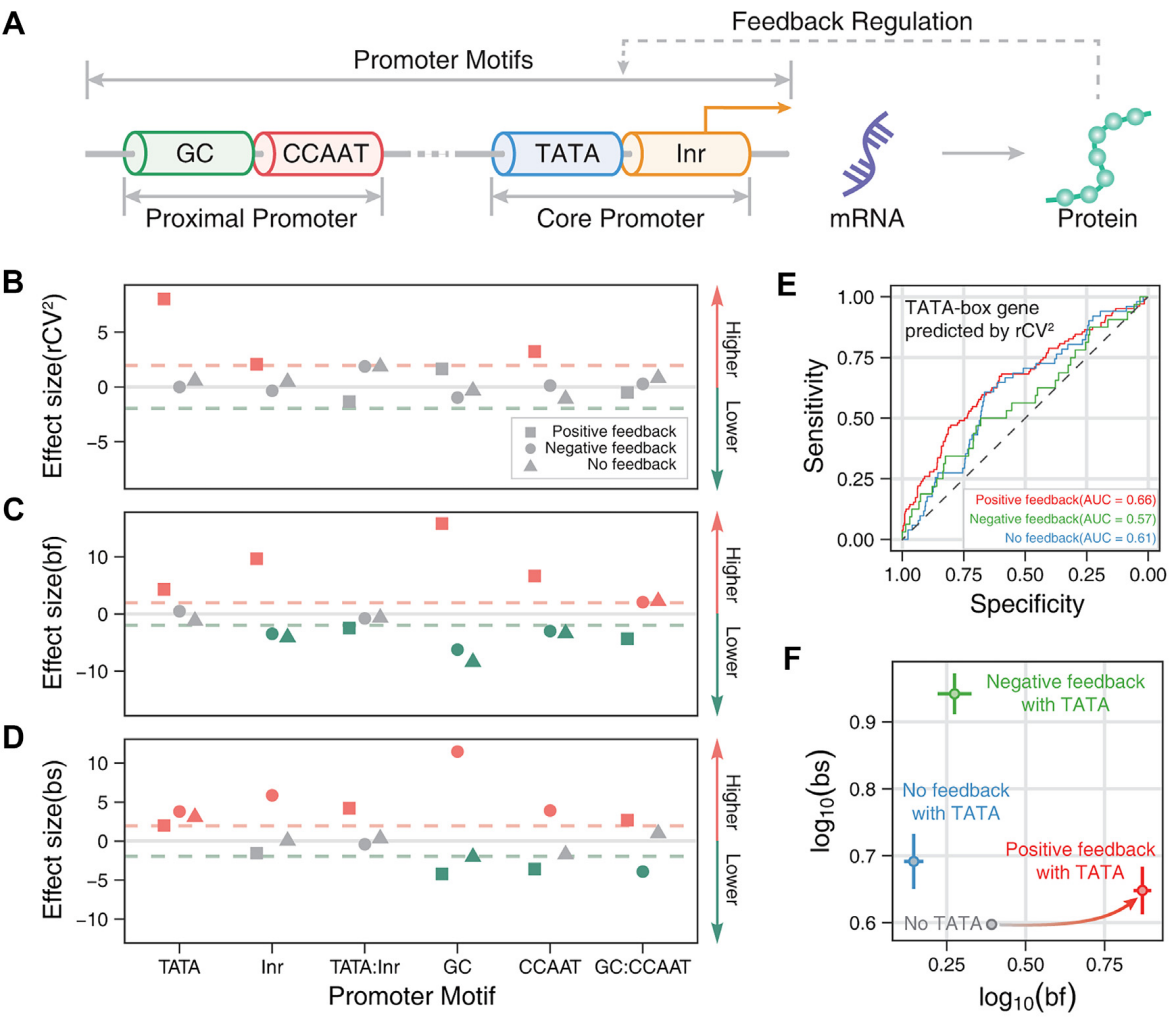
Genome-wide effects of promoter motifs on transcriptional burst kinetics in three cases of feedback regulation. (**A**) Schematic for a gene model that considers feedback regulation and promoter motifs (such as initiator, TATA-box, CCAAT-box, and GC-box). (**B–D**) Dependences of variability (rCV^2^, B), burst frequencies (bf, C) and burst sizes (bs, D) on promoter motifs for different feedback regulations, obtained through linear regressions. Each symbol shows the *t*-value in a multivariate linear regression model, which is used to judge whether to reject the null hypothesis (i.e. the feature does not correlate with the dependent variable). Color: significantly higher (red symbol), significantly lower (green symbol), and no apparent effect (gray symbol). Different symbols stand for different feedbacks: square for positive feedback, circle for negative feedback, and triangele for non-feedback. (**E**) ROC curves are used to distinguish the genes with TATA boxes according to the relative rCV^2^ rank. AUC is the area under the ROC curves. (**F**) Scatter plots of mean burst frequencies and mean burst sizes among the genes without TATA (gray), with positive feedback and TATA (red), with negative feedback and TATA (green), and without feedback but with TATA (blue). The solid lines near the scatter are error bars.

We first identified promoter motifs (TATA box, initiator, GC-box, and CCAAT-box) of each gene from the EPD database (54) (see ‘Materials and Methods’) (Figure 4A). Then, we found that both the TATA box and initiator positively regulated mean transcriptional levels, in line with the results obtained in previous studies (74) (Supplementary Figure S10a). Besides, we verified that the TATA genes with positive feedback had higher proportions than those genes with negative feedback or without feedback, whereas the other promoter motifs were uncorrelated to feedback forms (Supplementary Figure S10b). These results implied that the TATA box was a critical promoter motif for the regulation of transcription by a positive feedback mechanism, which might be supported by the following experimental observation: TATA boxes were enriched in the promoters of genes with fewer transcriptional pauses (75), and the TATA box sequence was specifically bound by the TATA-binding proteins that acted as general transcription factors to facilitate the localization of RNA polymerase II and transcription (76,77).

To investigate the genome-wide effects of promoter motifs on burst kinetics in the presence of feedback regulations, we performed multivariate statistical analysis using linear regression models (Figure 4B–D, see ‘Materials and Methods’). We also observed the Simpson's paradox that the effect of promoter motifs on variability and burst kinetics is different between distinguishing feedback regulation and without distinguishing feedback regulation.

First, we studied gene-expression variability. We characterized this variability with the residual squared coefficient of variation (*r*CV^2^) (see ‘Materials and Methods’) since this coefficient can disentangle the correlation of the CV^2^ and the average expression levels across cells (Supplementary Figure S9b). Therefore, we focus on *r*CV^2^ instead of CV^2^. By performing the linear regression of *r*CV^2^ (see ‘Materials and Methods’), we found the synergy between positive feedback and the TATA box (or initiator or CCAAT box) can amplify the expression variability (Figure 4B). This result was actually an extension of the previous result that the TATA box enlarged the gene-expression variability when feedback regulations were not distinguished (Supplementary Figure S10c) (13,78). As an additional evaluation, we used the *r*CV^2^ rank to predict the presence of the TATA-box and showed that the area under the ROC (receiver operating characteristic) curve, denoted by AUC, was larger in the case of positive feedback than in the case of negative feedback or non-feedback (Figure 4E), indicating that TATA boxes led to the larger gene-expression variability in the former case.

Next, we assessed burst frequencies and sizes. Similar to the case of expression variability, we also performed multivariate linear regression analyses on them. When feedbacks were not distinguished, we showed that TATA boxes significantly boosted burst frequencies of the genes (Supplementary Figure S10d). However, when considering different feedback forms, we observed that only TATA genes with positive feedback increased burst frequencies (Figure 4C). In addition, we observed that other promoter motifs had different degrees of effect on burst frequency, depending on feedback forms. These results were masked without distinguishing feedback forms (Supplementary Figure S10d). For burst sizes, it was reported that the genes with TATA box or initiator had larger burst sizes than those without TATA box or without initiator (16). We reproduced similar results (Supplementary Figure S10e), but observed that the TATA genes were expressed with larger burst sizes, independently of feedback regulation, and the genes with initiator had larger burst sizes only in the case of negative feedback (Figure 4D). GC-box and CCAAT-box on the distal promoter had opposite effects on burst sizes in the cases of positive and negative feedback (Figure 4D). In particular, no difference was found for all the genes if feedback forms were not distinguished (Supplementary Figure S10e).

Briefly, the above results indicated that the TATA box played a pivotal role in transcriptional bursting. It worked as a static promoter element to up-regulate burst sizes and simultaneously utilized a dynamic positive feedback regulation mechanism to increase burst frequencies (Figure 4F).

### Feedback regulations concealed the effects of TSS distribution on transcriptional burst kinetics

TSS can be divided into two classes according to its distribution: single TSS (sharp promoter) and multiple TSSs (broad promoter), both being important for gene expression (79,80). It was reported that the shapes of TSS distribution correlated with the category of genes, such as housekeeping genes and cell-type-specific genes, both exhibiting different transcriptional burst patterns (81). On the other hand, some experimental results indicated that feedback can regulate transcriptional initiation (23,82,83). A question naturally arose: how do the shapes of TSS distribution affect transcription burst kinetics in the presence of feedback regulation?

**Figure 5.**
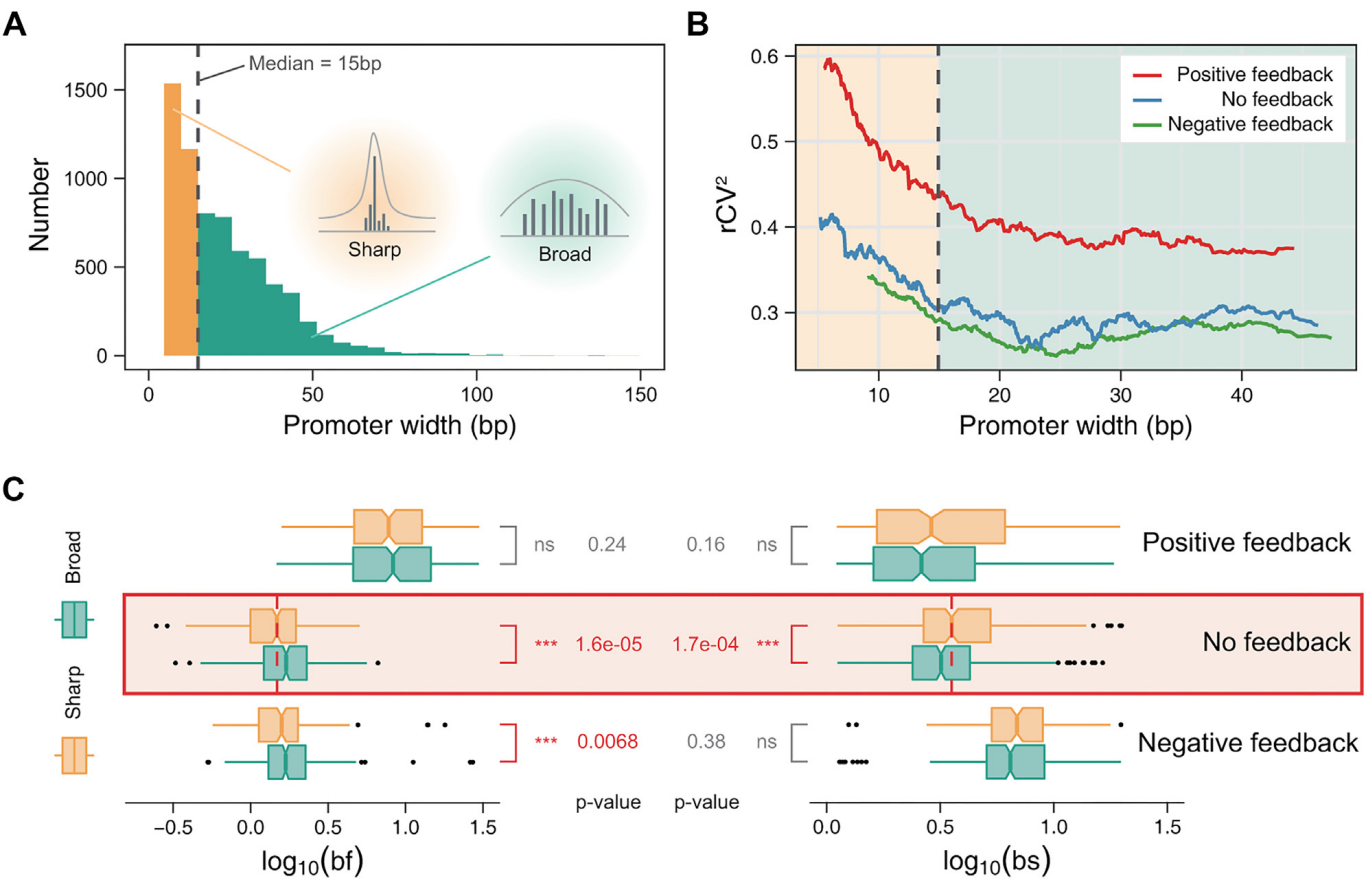
Genome-wide effects of TSS distributions on transcriptional burst kinetics in three cases of feedback regulation. (**A**) Histogram of genes, which are divided into two groups (sharp and broad) based on the median (dashed line) of promoter widths. (**B**) Changing trends of variability (*r*CV^2^) as a function of promoter width in three cases of feedback regulation: positive (red), negative (green) and non-feedbacks (blue). The left-hand side of the dashed line stands for sharp promoters (yellow region) and the right-hand side for broad promoters (green region). (**C**) Boxplots of burst frequencies (left) and burst sizes (right), where yellow squares stands for sharp promoters and green squares for broad promoters. *P*-values are indicated, and ns is the abbreviation of no significance.

To address this question, we used the R package CAGEr (55) to read CAGE data of FANTOM5 MEF cell (see ‘Materials and Methods’) and classified the promoters into ‘broad’ and ‘sharp’ ones (79) according to the median (15bp) of the widths of all sampled promoters as depicted in Figure 5A. Similarly, the influence of the TSS distribution on variability and burst kinetics was subject to Simpson's paradox in the case of with and without distinguishing feedback regulations.

We showed that the impacts of different TSS distributions on the mean expression level did not exhibit apparent differences in three cases of feedback regulation and all genes (Supplementary Figure S11a,b). This property can avoid possible errors in evaluating the expression variability (*r*CV^2^). Consistent with the observations in previous experimental studies (13), sharp promoters resulted in a significantly higher expression variability than broad promoters, independent of feedback forms (Supplementary Figure S11c, d). The *r*CV^2^ declined with increasing the width (< 15bp) of ‘sharp’ promoters but was almost unchanged with increasing the width of ‘broad’ promoters (Figure 5B). Notably, the curve of *r*CV^2^ vs. promoter width for the positive-feedback genes was always above that for the genes with negative feedback or non-feedback (Figure 5B).

Next, we investigated whether different TSS distributions affected burst frequencies and burst sizes differently. Although genes with ‘sharp’ promoters led to a higher expression variability than those with ‘broad’ promoters for arbitrary feedback forms, burst frequencies and sizes regulated by TSS distributions can exhibit significant discrepancy only in the absence of feedback (Figure 5C, Supplementary Figure S11e, f). Broad promoters led to higher burst frequencies and smaller burst sizes than sharp promoters (Figure 5C, red box), in agreement with the experimental observation that broad promoters tended to occur in the case of low RNA polymerase II pause, whereas sharp promoters tended to occur in the case of high RNA polymerase II pause (84–86). These results implied that on the genome-wide scale, feedback regulations significantly weakened the impacts of TSS distributions on transcriptional burst kinetics.

### E–P interactions mainly modulate burst frequencies only in the presence of positive feedbacks

**Figure 6.**
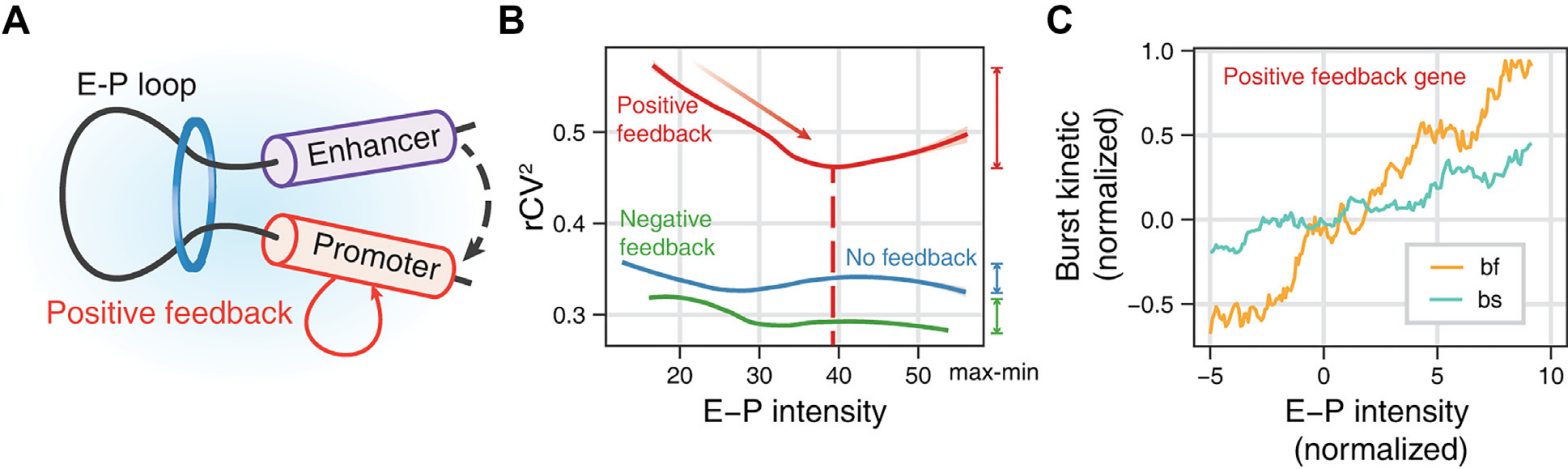
Genome-wide effects of enhancer–promoter interactions on transcriptional burst kinetics in three cases of feedback regulation. (**A**) Illustration of the E–P interaction with a positive feedback loop. (**B**) Dependence of noise (*r*CV^2^) on E–P interaction intensity for different feedback forms, where the dashed line represents the valley in case of positive feedback and the line segment on the right-hand side of the picture represents the maximum minus the minimum, that is, the amplitude of affecting the variability. Color: positive (red line), negative (green line), and non-feedbacks (blue line). (**C**) Dependences of normalized burst frequencies and sizes on E–P interaction intensity in the case of positive feedback.

Enhancers, DNA sequences located upstream of the promoter, are important regulators of eukaryotic development (87). Several lines of experimental evidence supported that E–P interactions (Figure 6A) may facilitate gene transcription (88–91) and can regulate transcriptional burst kinetics (14,16,92–95). In addition, some studies showed that enhancer and promoter activations might require positive and negative feedback regulations, each contributing the elements of the protein complement required for activation of other genes (96). These results raise important questions: does the genome-wide control of burst kinetics by E–P interactions involves feedback regulations? If so, how do feedbacks affect burst kinetics?

To address these questions, we first recovered the intensities of E–P interactions from (16) and performed LOESS regression. With the involvement of feedback regulations, the modulation of variability and transcriptional burst kinetics by E–P interactions also presents Simpson's paradox. We then showed that, for all genes, increasing the E–P intensities led to the rise of mean gene-expression levels (Supplementary Figure S12d) but to the decline of variability (*r*CV^2^) (Supplementary Figure S12e), indicating that stronger enhancers raised the expressions levels but lowered cell-to-cell variability in contrast to weaker enhancers (97). However, when distinguishing genes by feedback types, this pattern only appears in the case of positive feedback (Figure 6B, Supplementary Figure S12a), implying the important role of positive feedback in E–P interactions.

Next, we focused on burst frequencies and sizes. Previous molecular experiments and genome-wide inferences from scRNA-seq data showed that burst frequencies and sizes increased with promoting E–P interactions (93,94), and that enhancers mainly controlled burst frequencies (14,16,92–95,98–100). The same conclusion was obtained when we performed analysis without distinguishing feedback types (Supplementary Figure S12f, g). Notably, when the feedback types was considered, we found that as the E–P intensity increased, changes in burst frequencies and sizes were most apparent in the case of positive feedback (Supplementary Figure S12b,c). Moreover, the slope of the line for the dependence of burst frequencies on E–P intensity was larger than that for the dependence of burst sizes on E–P intensity (Figure 6C). In addition, we observed that this regulation effect of enhancers was saturated when the E–P interaction intensity exceeded a threshold (∼40) (Figure 6B and Supplementary Figure S12a-c). This result indicated that the function of the enhancer was not unlimited, in agreement with the theoretical prediction in our previous study (https://doi.org/10.1101/2022.01.24.477520).

The above genome-wide results provided direct support for the fact that the control of burst kinetics by E–P interactions was constrained by positive feedback regulations, in accordance with previous experimental results for a small number of genes (30,101).

## DISCUSSION

As the core process of life, gene transcription occurs stochastically, leading to variability in the mRNA and further protein abundances. This variability is believed to be mainly attributed to transcriptional bursting, a phenomenon that occurs commonly in both prokaryotes and eukaryotes. From the viewpoint of biophysics, the sources of transcriptional bursting are multilevel and multiscale (1). In this study, we have developed a statistical framework of the model-driven and data-driven integration to infer dynamic feedback regulations and transcriptional bursting kinetics from static scRNA-seq data, using a mechanistic mathematical model as the connecting thread.

The mechanistic model used in our inference framework was interpretable. It captured the scRNA-seq measurement process and the molecular mechanisms of transcriptional bursting processes. We showed that not only burst frequencies and sizes as well as expression variability but also feedback forms can be effectively and robustly inferred to explain biophysical phenomena, which were masked in the scRNA-seq data. Meanwhile, our inference method made the interpretable model tractable. We utilized the Gauss-Laguerre Quadrature Rules instead of the classical MCMC method to compute mRNA distribution with a high-order integral that is difficult to solve, thus making our scalable inference applicable on genome-wide scales. Our statistical inference framework laid a solid foundation for exploring the molecular mechanisms of stochastic gene expression based on single-cell data.

Our inference method provided a powerful tool for analyzing the joint effects of feedback regulation and promoter architecture and for revealing the genome-wide mechanisms of transcriptional burst kinetics. First, we found that at the same gene-expression levels, positive-feedback genes exhibited significantly higher gene-expression variability and higher burst frequencies as well as smaller burst sizes than negative-feedback genes on genome-wide scales. This finding indicated that different regulatory networks played distinct roles in modulating transcriptional burst kinetics (10). Second, we revealed that the TATA box, apart from being indicatives of enlarging the expression variability and raising burst sizes as suggested in previous studies (13,16), can utilize a positive feedback mechanism to increase burst frequencies. This result may explain the phenomenon that the RNA polymerase II on the TATA box gene had better localization and fewer transcriptional pauses (75,76). Third, broad promoters with multiple TSSs led to higher burst frequencies and smaller burst sizes, which were concealed by the feedback regulations. Finally, we showed that enhancer–promoter interactions modulated burst kinetics and primarily controlled burst frequency in the presence of positive feedback. All these results were obtained under the hidden hypothesis that the intrinsic behaviors of the different gene were statistically identical. Overall, these genome-wide evidences indicated that transcriptional burst kinetics was not only encoded by static promoter architectures but also constrained by dynamic gene regulatory networks.

Our inference framework based on the model-driven and data-driven combination was an extensible one for studying the general principles of transcriptional bursting. First, gene expression variability caused by transcriptional bursts comes not only from technical noise and feedback regulation as described in our hierarchical model, but also from many other potentially complex mechanisms, such as RNA polymerase II recruitment and pause release (102–105), alternative splicing (106,107), post-transcriptional regulations via mRNA degradation (108) and nuclear retention (109), chromatin movement (110), etc. (111–114), which all may affect burst kinetics. Second, promoter architecture can be described by a multi-state model since a transcription process would involve many molecular steps (115,116). It is unclear whether the multi-state architecture is more descriptive than the two-state model. Determining the number of gene states and studying the effect on burst kinetics is a long-term effort. Third, our hierarchical model only considered self-regulatory feedback (117), the simplest feedback form. More complex regulatory forms may exist in gene-expression systems (118). However, since they reflect high-level structure regulation (10), more complex yet reasonable mathematical models and more powerful inference methods need to be developed for better studying transcriptional burst kinetics. Fourth, most of the traditional models of gene expression were based on the Markov hypothesis (69,119). In organisms, however, the processes of molecular synthesis may be non-Markovian, and increasing time-resolved data have verified the extensive existence of molecular memory (120,121). Therefore, it is necessary to extend Markov models to non-Markov ones (122–124). But this is a great challenge to numerical solutions and statistical inferences. Finally, we point out that choosing a suitable model involves trade-off problems since more complex models would bring less consensus on general principles of transcriptional bursting (4).

Finally, studying transcriptional burst kinetics may start with a data-driven approach as done in our statistical inference framework. Our predictions of burst kinetics using scRNA-seq data were based on the assumption that the abundances of mRNA and protein were highly dependent (65). Recently, more and more studies of sequencing methods have paid attention to measuring the profiles of multi-type molecules in single-cell levels, such as simultaneous quantification of intracellular mRNA and protein (125), which can better describe cell states (126). For feedback loop types our method predicted, we found that many genes have been confirmed by biological experiments (Supplementary Table S1). Moreover, the identification of feedback loops can be more convincing by using multimodal data combined with scRNA-seq such as ENCODE (127) and some automated packages (128). In addition, time-resolved data can provide more information compared to static data. We believe that with the continuous progress in measurement technologies, time-resolved single-cell data will be primary means to study the transcription burst kinetics in the future (https://doi.org/10.1101/2022.06.19.496754). Meanwhile, spatial transcriptome multimodal data (129–132) and chromatin structural data (133) provided good opportunities for in-depth studies of burst kinetics. Analysis of those multimodal single-cell data or integrated data can help us discover more credible biological knowledge but would also bring challenges for developing statistical methods to infer dynamic molecular mechanisms masked in static single-cell data.

## DATA AVAILABILITY

All the analysis results and inference code that support the findings of this study are provided through https://github.com/cellfate/BurstFeedback or https://zenodo.org/record/7371318 (DOI: 10.5281/zenodo.7371318).

## Supplementary Material

gkac1204_Supplemental_File
