# Supplementary material for: Genome-wide inference reveals that feedback regulations constrain promoter-dependent transcriptional burst kinetics
Source: Nucleic Acids Res. 2022 Dec 30;51(1):68–83. doi: 10.1093/nar/gkac1204 (PMC9874261; doi:10.1093/nar/gkac1204)
Supplement: gkac1204_Supplemental_File [file gkac1204_supplemental_file.pdf]

Supplementary File

Genome-wide inference reveals that feedback regulations constrain promoter-dependent transcriptional burst kinetics

Songhao Luo[1,2,†], Zihao Wang[1,2,†], Zhenquan Zhang[1,2], Tianshou Zhou[1,2,*], and Jiajun Zhang[1,2,*]

[1] Guangdong Province Key Laboratory of Computational Science, Sun Yat-sen University, Guangzhou 510275, P. R. China

[2] School of Mathematics, Sun Yat-sen University, Guangzhou, Guangdong Province, 510275, P. R. China

[†] These authors contributed equally to this work.

[*] To whom correspondence should be addressed.

Email: mcszhtsh@mail.sysu.edu.cn, zhjiajun@mail.sysu.edu.cn

Content

1. Supplementary Text **3**

1.1 Hierarchical model 3

Measurement model 3

Gene expression model 4

Hierarchical model for autoregulatory feedback system 7

1.2 Telegraph model 8

2. Supplementary Figures **10**

3. Supplementary Table **22**

4. Reference **25**

1. Supplementary Text

1.1 Hierarchical model

Observed scRNA-seq counts are a noisy reflection of true gene-expression levels. We model the observed counts as a hierarchical model (Supplementary Figure S1a), which captures two core mechanistic processes: a sequencing measurement process (Supplementary Figure S1b) and a gene expression process (Supplementary Figure S1c). To make this idea precise, we let n denote the true number of mRNAs expressed in cells, following the stationary distribution $P_{\text{gene}}(n)$ from a specific gene expression system. Let y denote the observed number of mRNA molecules in cells, which in principle follows a conditional distribution denoted by $P_{\text{meas}}(y|n)$. Thus, the framework of the hierarchical model can be mathematically described as

$$\begin{aligned} P_{\text{obs}}(Y=y;\cdot) &= P_{\text{meas}}(y|n) \underset{n}{\wedge} P_{\text{gene}}(n) \\ &= \int_0^\infty P_{\text{meas}}(y|n) P_{\text{gene}}(n) dn \end{aligned} \tag{S1}$$

where the $\wedge$ notation represents the mixtures of distributions. Next, we rationalize our hierarchical model through the following detailed analyses.

Measurement model

First, we can couple a sequencing process into a gene expression model via the measurement model $P_{\text{meas}}(y|n)$ of scRNA-seq data (1). This is because the reads we observed are actually sampled from the true number of mRNAs by sequencing instruments. Assume that the observed reads of scRNA-seq data are the sampling result of mRNA without replacement, and that we can use a hypergeometric distribution to describe the measurement process (Supplementary Figure S1b). In general, hypergeometric distribution is computationally challenging, but we can use a binomial distribution to approximate it if the sample number is much smaller than the total number, i.e.,

$$Y \sim P_{\text{meas}}(y|n) = \text{Binomial}(n, \lambda), \tag{S2}$$

where $\lambda \in [0,1]$ is the sampling probability in the sequencing process.

Note that a discrete gene expression model can be written in a Poisson representation, that is,

$$\begin{aligned} P_{\text{gene}}(n) &= \int_0^\infty \text{Poisson}(n|x) f(x) dx \\ &\triangleq \text{Poisson}(n|x) \underset{x}{\wedge} f(x). \end{aligned} \tag{S3}$$

where $f(x)$ is a kernel density function. Then, we can use the following hierarchical model to describe the observed number by taking a binomial distribution as a measurement model (Eq. (S2)),

$$Y \sim \text{Binomial}(n,\lambda) \underset{n}{\wedge} \text{Poisson}(n|x) \underset{x}{\wedge} f(x). \tag{S4}$$

Note that for the mixture distribution $\text{Binomial}(n,\lambda) \underset{n}{\wedge} \text{Poisson}(n|x)$, we can derive its probability-generating function

$$\begin{aligned} G(z) &= \sum_{i=0}^{\infty}\sum_{n=0}^{\infty} \frac{n!\lambda^i (1-\lambda)^{n-i} e^{-x} x^n z^i}{i!(n-i)!n!} \\ &= \sum_{n=0}^{\infty} (1-\lambda+\lambda z)^n \frac{e^{-x} x^n}{n!} \\ &= e^{x(1-\lambda+\lambda z)-x} = e^{\lambda x(z-1)}, \end{aligned} \tag{S5}$$

which is the probability generating functions of $\text{Poisson}(\lambda x)$. Thus, Eq. (S4) can be rewritten as

$$Y \sim \text{Poisson}(y|\lambda x) \underset{x}{\wedge} f(x) \tag{S6}$$

which is actually a Poisson representation for scRNA-seq data with a factor λ.

Gene expression model

In this section, we discuss a discrete gene-expression model (described by $P_{\text{gene}}(n)$) that can simultaneously characterize transcriptional burst kinetics and feedback regulations. Since transcription factors (TFs) are often taken as feedback regulators, a gene expression model with feedback should consider protein levels. However, techniques for genome-wide measurement of proteomics are not yet sufficient for this consideration. An alternative approach is to use high-throughput scRNA-seq data to replace the protein levels (Supplementary Figure S1c) (2), and this is reasonable since the abundances of mRNA and protein are highly related (3). In the following, we rationalize that scRNA-seq data are suitable for the inference of feedback type by using Eq. (4) in the main text.

First, let us consider a transcription model with feedback regulation, described by the following reaction scheme

$$\begin{aligned} &\text{OFF} \xrightarrow{k_{\text{on}}(n)} \text{ON}, \\ &\text{ON} \xrightarrow{k_{\text{off}}} \text{OFF}, \\ &\text{ON} \xrightarrow{k_{\text{syn}}} \text{ON} + \text{mRNA}, \\ &\text{mRNA} \xrightarrow{k_{\text{deg}}} \varnothing. \end{aligned} \tag{S7}$$

where k_{on} and k_{off} are the promoter's activation and inactivation rates respectively, k_{syn} and k_{deg} are the mRNA's synthesis and degradation rates respectively. Here we consider that only the promoter activation rate is auto-regulated by TFs (4,5), and assume that the number of proteins (TFs) is proportional to the number of mRNAs, i.e., the number of proteins is $r \cdot n$, where n is the mRNAs numbers and r is a constant ratio factor. The regulation of the activation process by proteins is characterized by a Hill function. Let $P_{\text{off}}(n,t)$ and $P_{\text{on}}(n,t)$ be the probability that there are n mRNAs in OFF state and ON state at time t, respectively. We then write two chemical master equations (CMEs) for Eq. (S7)

$$\begin{aligned}\frac{\partial P_{\text{off}}(n,t)}{\partial t} &= -k_{\text{on}}\left[\frac{K^h}{K^h+(rn)^h}+\varepsilon\right]P_{\text{off}}(n,t)+k_{\text{off}}P_{\text{on}}(n,t)\\ &\quad +k_{\text{deg}}\left[(n+1)P_{\text{off}}(n+1,t)-nP_{\text{off}}(n,t)\right],\\ \frac{\partial P_{\text{on}}(n,t)}{\partial t} &= k_{\text{on}}\left[\frac{K^h}{K^h+(rn)^h}+\varepsilon\right]P_{\text{off}}(n,t)-k_{\text{off}}P_{\text{on}}(n,t)\\ &\quad +k_{\text{deg}}\left[(n+1)P_{\text{on}}(n+1,t)-nP_{\text{on}}(n,t)\right]\\ &\quad +k_{\text{syn}}\left[P_{\text{on}}(n-1,t)-P_{\text{on}}(n,t)\right].\end{aligned} \tag{S8}$$

where K denotes the dissociation constant between the promoter and proteins, and $\varepsilon = k_{\varepsilon}/k_{\text{on}}$ with k_{ε} being the basal activation rate represents a basal burst rate in the absence of regulation. Note that the Hill coefficient $h>0$ corresponds to negative feedback, and $h<0$ to positive feedback.

Next, we find the stationary mRNA distribution $P_{\text{gene}}(n)=\lim_{t\to\infty}\left[P_{\text{off}}(n,t)+P_{\text{on}}(n,t)\right]$ by introducing a Poisson representation. For this, we define two kernel density functions $f_{\text{off}}(x,t)$ and $f_{\text{on}}(x,t)$ according to the following relationships

$$\begin{aligned}P_{\text{off}}(n,t) &= \int_0^{x_{\max}}\frac{x^n e^{-x}}{n!}f_{\text{off}}(x,t)\,dx,\\ P_{\text{on}}(n,t) &= \int_0^{x_{\max}}\frac{x^n e^{-x}}{n!}f_{\text{on}}(x,t)\,dx,\end{aligned} \tag{S9}$$

where $x_{\max}$ is the maximum of the maximum values of functions $f_{\text{off}}(x,t)$ and $f_{\text{on}}(x,t)$ with regard to x. Note that the total kernel density function $f(x,t)=f_{\text{off}}(x,t)+f_{\text{on}}(x,t)$ satisfies the normalization condition $\int_0^{x_{\max}} f(x,t)\,dx=1$. Substituting Eq. (S9) into Eq. (S8), multiplying n on both sides of Eq. (S8), and summing up n from 0 to infinity approximately, we obtain the following differential Chapman-Kolmogorov equations for $f_{\text{off}}(x,t)$ and $f_{\text{on}}(x,t)$,

$$\frac{\partial f_{\text{off}}(x,t)}{\partial t} = -k_{\text{on}}\left[\frac{K^h}{K^h+(rx)^h}+\varepsilon\right] f_{\text{off}}(x,t) + k_{\text{off}} f_{\text{on}}(x,t) - \frac{\partial}{\partial \lambda} J_{\text{off}}(x;t),$$
$$\frac{\partial f_{\text{on}}(x,t)}{\partial t} = k_{\text{on}}\left[\frac{K^h}{K^h+(rx)^h}+\varepsilon\right] f_{\text{off}}(x,t) - k_{\text{off}} f_{\text{on}}(x,t) - \frac{\partial}{\partial \lambda} J_{\text{on}}(x;t), \tag{S10}$$

with the no-flux boundary conditions $J_{\text{off}}(0;t) = J_{\text{off}}(x_{\max};t) = 0$, and $J_{\text{on}}(0;t) = J_{\text{on}}(x_{\max};t) = 0$, where $J_{\text{off}}(x;t) = -k_{\text{deg}} x f_{\text{off}}(x,t)$ and $J_{\text{on}}(x;t) = (k_{\text{syn}} - k_{\text{deg}} x) f_{\text{on}}(x,t)$. It is reasonable to choose $x_{\max} = k_{\text{syn}}/k_{\text{deg}}$ to make the boundary terms vanish. Thus, the boundary conditions imply that $f_{\text{off}}(k_{\text{syn}}/k_{\text{deg}}, t) = 0$ and $f_{\text{on}}(0,t) = 0$.

From Eq. (S10), we find that the stationary distribution $f(x) = \lim_{t\to\infty}\left[f_{\text{off}}(x,t) + f_{\text{on}}(x,t)\right]$ takes the form

$$f(\hat{x}) = C\hat{x}^{\tilde{k}_{\text{on}}(\varepsilon+1)-1}(1-\hat{x})^{\tilde{k}_{\text{off}}-1}\left(1+\left(r\hat{x}/\hat{K}\right)^h\right)^{-\tilde{k}_{\text{on}}/h}, \tag{S11}$$

where $\hat{x} = x/\tilde{k}_{\text{syn}}$, $\hat{K} = K/\tilde{k}_{\text{syn}}$, $\tilde{k}_{\text{syn}} = k_{\text{syn}}/k_{\text{deg}}$, $\tilde{k}_{\text{on}} = k_{\text{on}}/k_{\text{deg}}$, $\tilde{k}_{\text{off}} = k_{\text{off}}/k_{\text{deg}}$, and C is a normalization constant. Note that $\hat{x}$ represents a non-dimensional mRNA quantity normalized by $x_{\max}$.

Now, we consider the case of bursty expression (i.e., assuming $\tilde{k}_{\text{off}} \gg \tilde{k}_{\text{on}} > 1$) (4,6). In this case, function $f(\hat{x})$ can be approximated as

$$\begin{aligned} f(\hat{x}) &= C\hat{x}^{\tilde{k}_{\text{on}}(\varepsilon+1)-1}\left(1-\frac{\tilde{k}_{\text{off}}\hat{x}}{\tilde{k}_{\text{off}}}\right)^{\tilde{k}_{\text{off}}-1}\left(1+\left(r\hat{x}/\hat{K}\right)^h\right)^{-\tilde{k}_{\text{on}}/h} \\ &\approx \tilde{C}\hat{x}^{\tilde{k}_{\text{on}}(\varepsilon+1)-1}e^{-\tilde{k}_{\text{off}}\hat{x}}\left(1+\left(r\hat{x}/\hat{K}\right)^h\right)^{-\tilde{k}_{\text{on}}/h}. \end{aligned} \tag{S12}$$

where $\tilde{C}$ is a normalization constant. Therefore, the stationary mRNA distribution $P_{\text{gene}}(n)$ is given by

$$P_{\text{gene}}(n) = \int_0^1 \text{Poisson}\left(n\middle|\tilde{k}_{syn}\hat{x}\right) f(\hat{x})\,d\hat{x}. \tag{S13}$$

To verify the correctness of Eq. (S13) obtained from Eq. (S7), we use the mRNA distribution generated by the Gillespie algorithm to compare it with the theoretically approximate solution obtained by substituting Eq. (S11) or Eq. (S12) into Eq. (S13). From Supplementary Figure S1d-g, we observe that the approximate theoretical results fit well with the numerically generated data, whatever in positive and negative feedback cases. This indicates that the stationary solution of the discrete gene expression model (Eq. (S7)) can be well described by Poisson representation.

Noting that the relationship $\hat{x} = x/x_{\max} = x/\tilde{k}_{\text{syn}}$ and substituting Eq. (S12) into Eq. (S13), we find that the stationary distribution characterized by the burst parameters can be finally expressed as

$$P_{\text{gene}}(n)=\tilde{C}\int_0^{\infty}\text{Poisson}(n|x)\,x^{a(\varepsilon+1)-1}e^{-x/b}\left(1+(x/k)^h\right)^{-a/h}dx. \tag{S14}$$

where $a=\tilde{k}_{\text{on}}$ is burst frequency, $b=\tilde{k}_{\text{syn}}/\tilde{k}_{\text{off}}$ is burst size and $k=K/r$ is a parameter.

Hierarchical model for autoregulatory feedback system

Since the feedback system Eq. (S7) can be described by a Poisson representation, i.e., Eq. (S14), we can choose the binomial distribution given by Eq. (S2) as the measurement model. Using Eq. (S6), the observed mRNA expression distribution of scRNA-seq data is calculated according to

$$\begin{aligned}P_{\text{obs}}(y)&=\int_0^{\infty}\text{Poisson}(y|\lambda x)\,f(x)\,dx\\&=\tilde{C}\int_0^{\infty}\text{Poisson}(y|\lambda x)\,x^{a(\varepsilon+1)-1}e^{-x/b}\left(1+(x/k)^h\right)^{-a/h}dx.\end{aligned} \tag{S15}$$

From the above analysis, we can see that the hierarchical model described by Eq. (S15) is an interpretable and mechanistic one. Moreover, it can be used in the simultaneous inference of transcriptional burst kinetics and feedback (as shown in the main text) since we can extract five significant parameters $\theta=(a,b,\varepsilon,k,h)$ of the auto-regulatory feedback system from scRNA-seq data.

We emphasize that the kernel density function $f(x)$ in Eq. (S15) is in a form similar to the stationary distribution from (7), but the authors in that paper used a continuous stationary distribution to characterize translational bursting. In contrast, we use a hierarchical model, which combines the measurement process with a binomial distribution and the gene-expression process with a discrete stationary distribution (i.e., Eq. (S14)), to infer transcriptional bursting kinetics and feedback forms from scRNA-seq data.

1.2 Telegraph model

The classical telegraph model considers two gene states: an OFF state where the gene is not expressed and an ON state where the gene is transcribed. Specifically, the switching rates from OFF to ON and from ON to OFF are k_{on} and k_{off}, respectively. The gene transcription rate is k_{syn} and the mRNA degradation rate is k_{deg}. The model is described by the following four reactions:

$$\begin{aligned}&\mathrm{OFF}\xrightarrow{k_{\mathrm{on}}}\mathrm{ON}\\&\mathrm{ON}\xrightarrow{k_{\mathrm{off}}}\mathrm{OFF}\\&\mathrm{ON}\xrightarrow{k_{\mathrm{syn}}}\mathrm{ON}+\mathrm{mRNA},\\&\mathrm{mRNA}\xrightarrow{k_{\mathrm{deg}}}\varnothing.\end{aligned}\tag{S16}$$

Moreover, it is assumed that all the reaction events involved are Markovian, i.e., the probability that the reaction events happen depends only on the present state of the system, independent of the prior history.

Let $P(x;t)$ represent the probability that the mRNA has x molecules at time t. The steady-state distribution of these equations has been shown (8) to take the form

$$P(x)=\frac{\Gamma\left(\tilde{k}_{\mathrm{on}}+x\right)\Gamma\left(\tilde{k}_{\mathrm{on}}+\tilde{k}_{\mathrm{off}}\right)}{\Gamma\left(\tilde{k}_{\mathrm{on}}\right)\Gamma\left(\tilde{k}_{\mathrm{on}}+\tilde{k}_{\mathrm{off}}+x\right)}\frac{\tilde{k}_{\mathrm{syn}}^{x}}{x!}\,{}_1F_1\left(\tilde{k}_{\mathrm{on}}+x;\tilde{k}_{\mathrm{on}}+\tilde{k}_{\mathrm{off}}+x;-\tilde{k}_{\mathrm{syn}}\right),\tag{S17}$$

where the switching rates are in unit of mRNA decay rate, i.e., $\tilde{k}_{\mathrm{on}}=k_{\mathrm{on}}/k_{\mathrm{deg}}$, $\tilde{k}_{\mathrm{off}}=k_{\mathrm{off}}/k_{\mathrm{deg}}$, $\tilde{k}_{\mathrm{syn}}=k_{\mathrm{syn}}/k_{\mathrm{deg}}$. Moreover, the distribution in Eq. (S17) can be regarded as a Poisson-Beta distribution (9), i.e., the mixture of a Poisson distribution and a Beta distribution. Specifically, if the Poisson distribution has a mean $\tilde{k}_{\mathrm{syn}}t$, where $\tilde{k}_{\mathrm{syn}}$ is a scale parameter and t follows the Beta distribution with parameters $\left(\tilde{k}_{\mathrm{on}},\tilde{k}_{\mathrm{off}}\right)$, then the expression of $P(x)$ can be reproduced by calculating

$$P(x)=\int_0^1\frac{\left(\tilde{k}_{\mathrm{syn}}t\right)^x}{x!}e^{-\tilde{k}_{\mathrm{syn}}t}\frac{t^{\tilde{k}_{\mathrm{on}}-1}(1-t)^{\tilde{k}_{\mathrm{off}}-1}}{B\left(\tilde{k}_{\mathrm{on}},\tilde{k}_{\mathrm{off}}\right)}dt,\tag{S18}$$

In spite of this fact, calculating the integral in Eq. (S18) is time-consuming. Therefore, we exploit the Gauss-Jacobi quadrature method (10) to compute Eq. (S18). If changing the variable $u=2t-1$, Eq. (S18) can be rewritten as

$$P(x)=\frac{1}{B\left(\tilde{k}_{\mathrm{on}},\tilde{k}_{\mathrm{off}}\right)}\frac{1}{2^{\tilde{k}_{\mathrm{on}}+\tilde{k}_{\mathrm{off}}-1}}\int_{-1}^{1}\frac{\left(\tilde{k}_{\mathrm{syn}}(u+1)/2\right)^x}{x!}e^{-\tilde{k}_{\mathrm{syn}}(u+1)/2}(1+u)^{\tilde{k}_{\mathrm{on}}-1}(1-u)^{\tilde{k}_{\mathrm{off}}-1}du.\tag{S19}$$

Finally, based on the Gauss-Jacobi quadrature method and maximum likelihood estimation method,

we can estimate the model parameters $\left(\tilde{k}_{\text{on}}, \tilde{k}_{\text{off}}, \tilde{k}_{\text{syn}}\right)$ and compute the burst size $\tilde{k}_{\text{syn}} / \tilde{k}_{\text{off}}$, burst frequency $\tilde{k}_{\text{on}}^{-1}$ and variability of mRNA.

2. Supplementary Figures

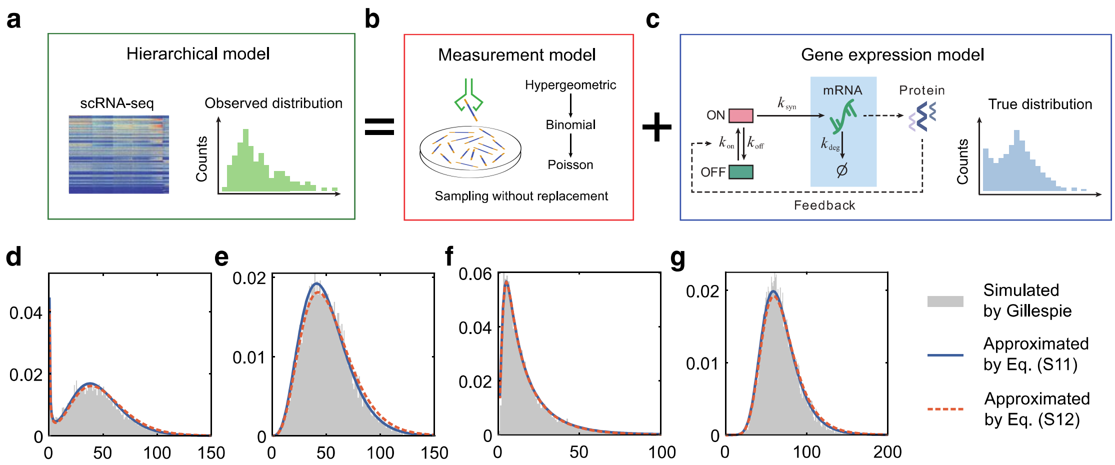

Supplementary Figure S1. Hierarchical model for generating single-cell data. (a) Hierarchical model is used in modeling the observed distribution of scRNA-seq data, which can be considered as a coupling of two mechanistic processes: measurement model (b) and gene expression model (c). **(b)** Schematic diagram of the measurement model. The sequencing process can be considered as the sampling without replacement for mRNA. Alternative models can be hypergeometric distributions or approximate Binomial distributions. **(c)** Schematic diagram of a gene-expression model. The proteins produced by mRNA translation can interact back with the gene promoter, thus affecting the rate of state switching. The shading indicates the transcriptome data used to replace protein abundance in the actual inference, under the assumption of linear dependence of mRNA and protein. And this model is used in modeling the 'true' distribution of gene expression. **(d-e)** Comparisons of the distributions of simulated (gray histogram) and two approximate theoretical results (The solid blue line corresponds to Eq. (S11), and the dotted orange line corresponds to Eq. (S12)). Parameters values in the case of positive feedback: (d) $k_{on} = 5$, $k_{off} = 100$, $k_{syn} = 1000$, $K/r = 10$, $h = -2$, $\varepsilon = 0.05$ and $k_{deg} = 1$; (e) $k_{on} = 5$, $k_{off} = 100$, $k_{syn} = 1000$, $K/r = 1$, $h = -4$, $\varepsilon = 0.05$ and $k_{deg} = 1$. Parameters values in the case of negative feedback: (f) $k_{on} = 10$, $k_{off} = 50$, $k_{syn} = 1000$, $K/r = 1$, $h = 2$, $\varepsilon = 0.05$ and $k_{deg} = 1$; (g) $k_{on} = 10$, $k_{off} = 50$, $k_{syn} = 1000$, $K/r = 50$, $h = 4$, $\varepsilon = 0.05$ and $k_{deg} = 1$.

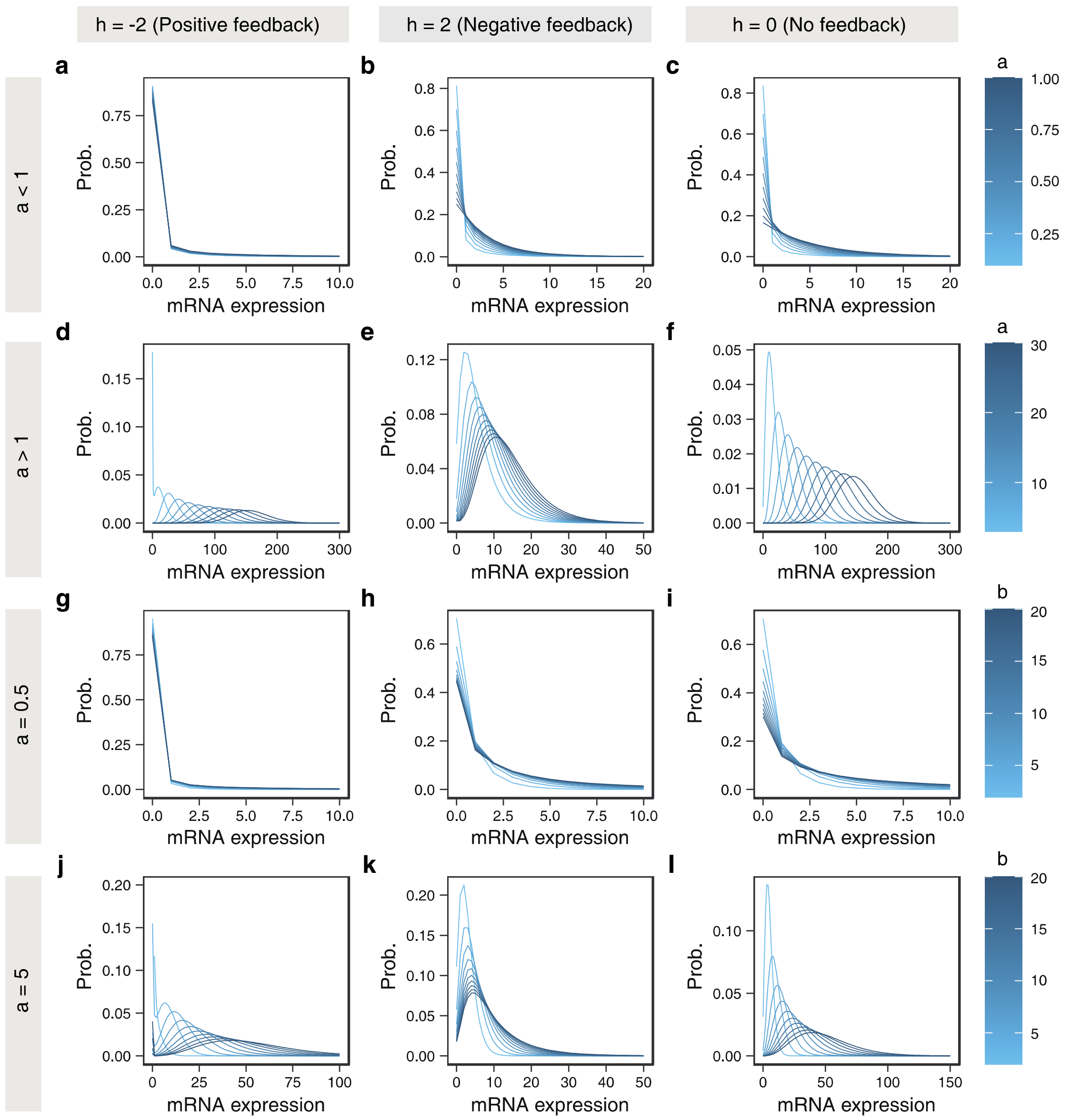

Supplementary Figure S2. Influences of parameters a and b on static mRNA distributions in the hierarchical model, where the left column is for positive feedback ($h=-2$), the middle column for negative feedback ($h=2$), and the right column for no feedback ($h=0$). **(a-f)** Static mRNA distribution for $a<1$ (a-c) and for $a>1$ (d-f) (see color bars), where the other parameter values are set as $b=10$, $\varepsilon=0.05$, $k=5$, $\lambda_g=0.5$. **(g-l)** Static mRNA distributions for fixed $a=0.5$ (g-i) and $a=5$ (j-l), where color bars show increases in b, and the other parameter values are set as $\varepsilon=0.05$, $k=5$, $\lambda_g=0.5$.

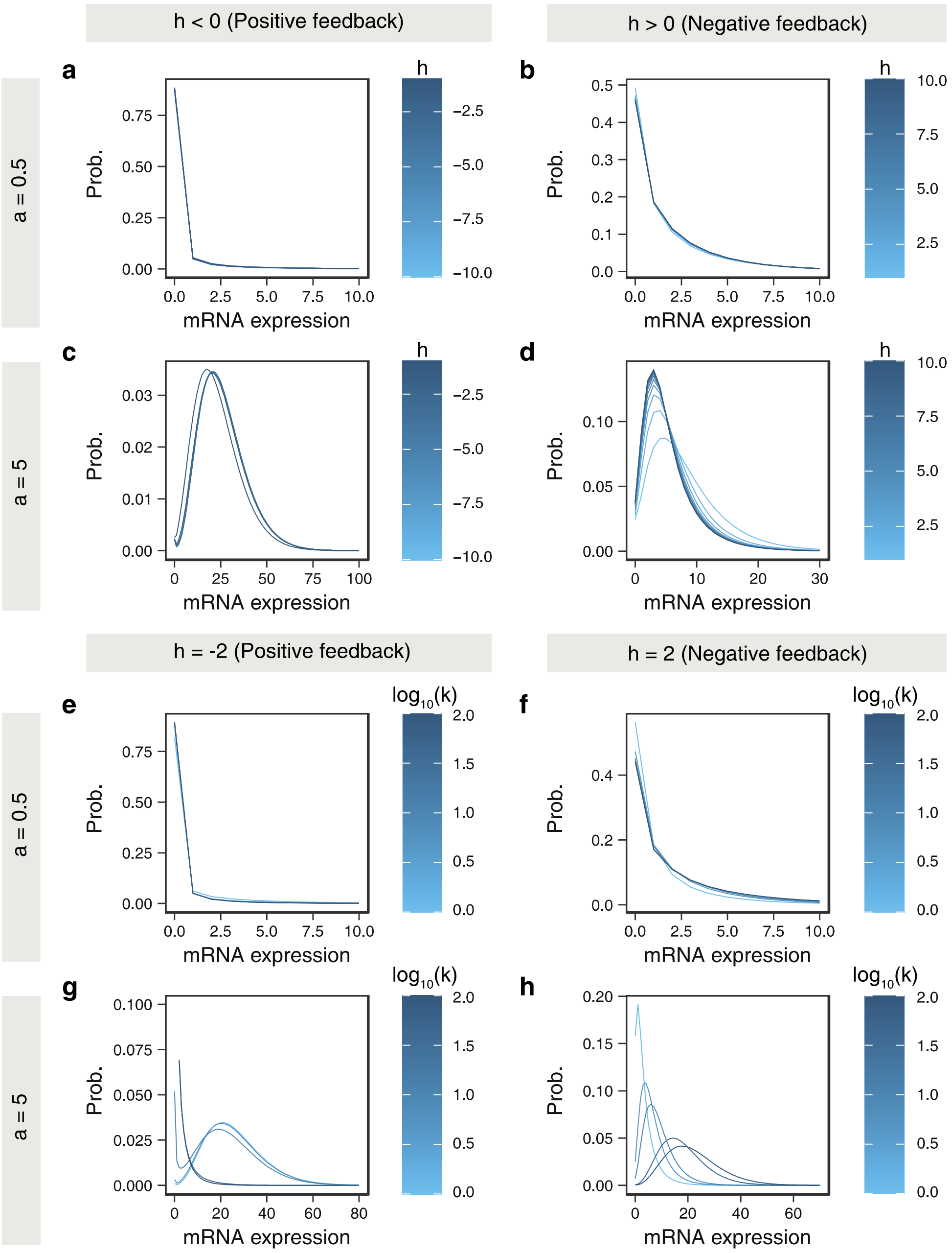

Supplementary Figure S3. Influences of parameters h and k on static mRNA distributions in the hierarchical model, where the left column is for positive feedback ($h = -2$), and the right column for negative feedback ($h = 2$). **(a-d)** Influences of feedback strengths (color bars) on static mRNA distribution for fixed $a = 0.5$ (a-b) and for fixed $a = 5$ (c-d), where the other parameter values are set as $b = 10$, $\varepsilon = 0.05$, $k = 5$, $\lambda_g = 0.5$. **(e-h)** Influences of equilibrium binding constant k (color bars) on static mRNA distribution, where (e-f) is for $a = 0.5$ and (g-h) for $a = 5$, and the other parameter values are set as: $\varepsilon = 0.05$, $k = 5$, $\lambda_g = 0.5$.

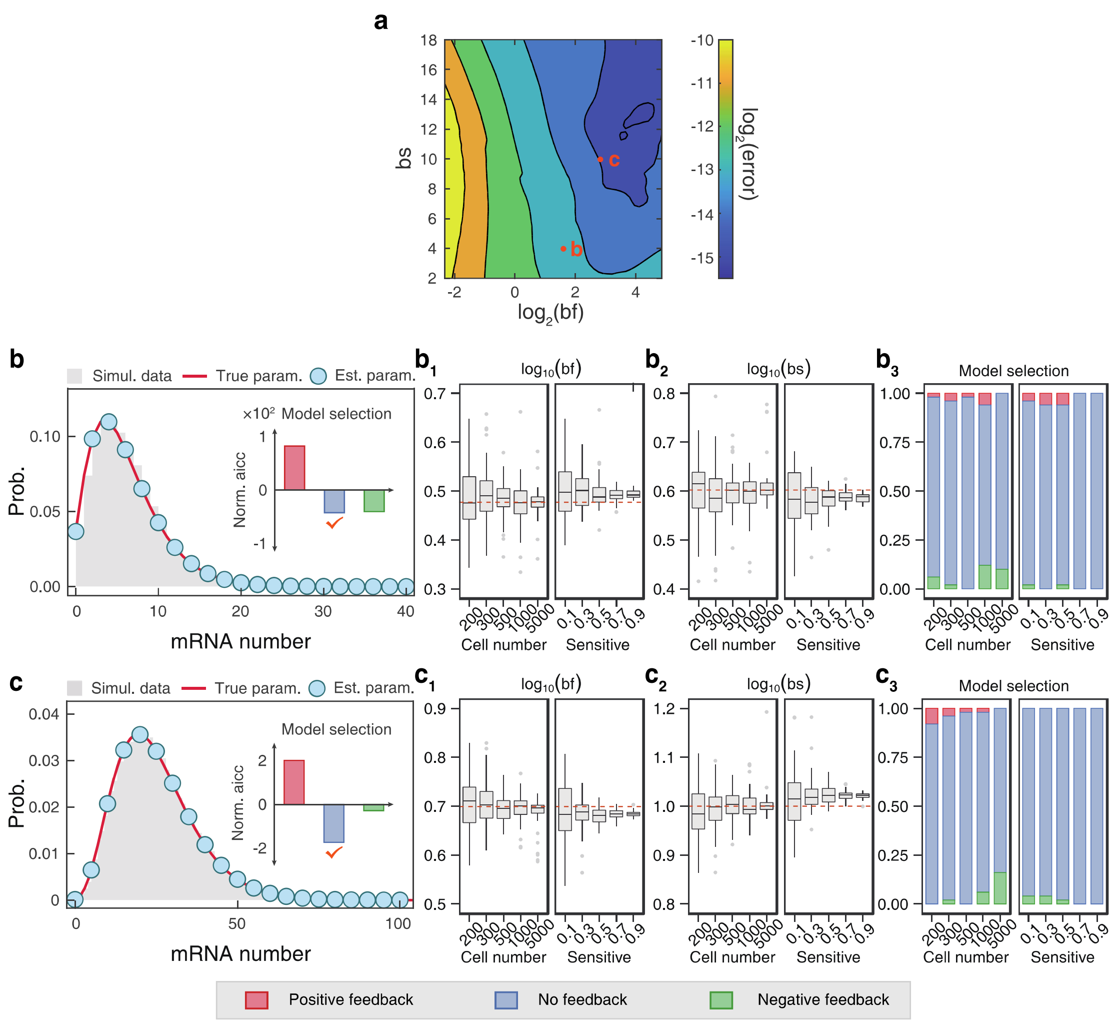

Supplementary Figure S4. Precision and robustness of inferred results in the case of no feedback. **(a)** Contours illustrate the error (color bars) of inferred burst kinetics from simulated expression data. The orange dots represent burst frequencies and sizes in the examples showed in (b) and (c). **(b)** mRNA distributions for fixed $a=3$, $b=4$, where the gray histograms are for simulated data, red solid line for the distribution generated using true kinetic parameters and blue points for the distribution generated using estimated kinetic parameters; Inset: analysis of model selection based on the corrected Akaike information criterion (AICc) that is normalized for visualization convenience, where three colored bars represent the results of model selection for three cases of different feedbacks and the check mark represents the final selected model. **(b_1-b_3)** Boxplot of inferred burst frequencies, burst sizes and feedback models as functions of different cell numbers and random deletions (sensitive), where the red dashed line corresponds to the true parameter. **(c and c_1-c_3)** Analysis of robustness inference similar to b and b_1-b_3 for fixed $a=5$, $b=10$.

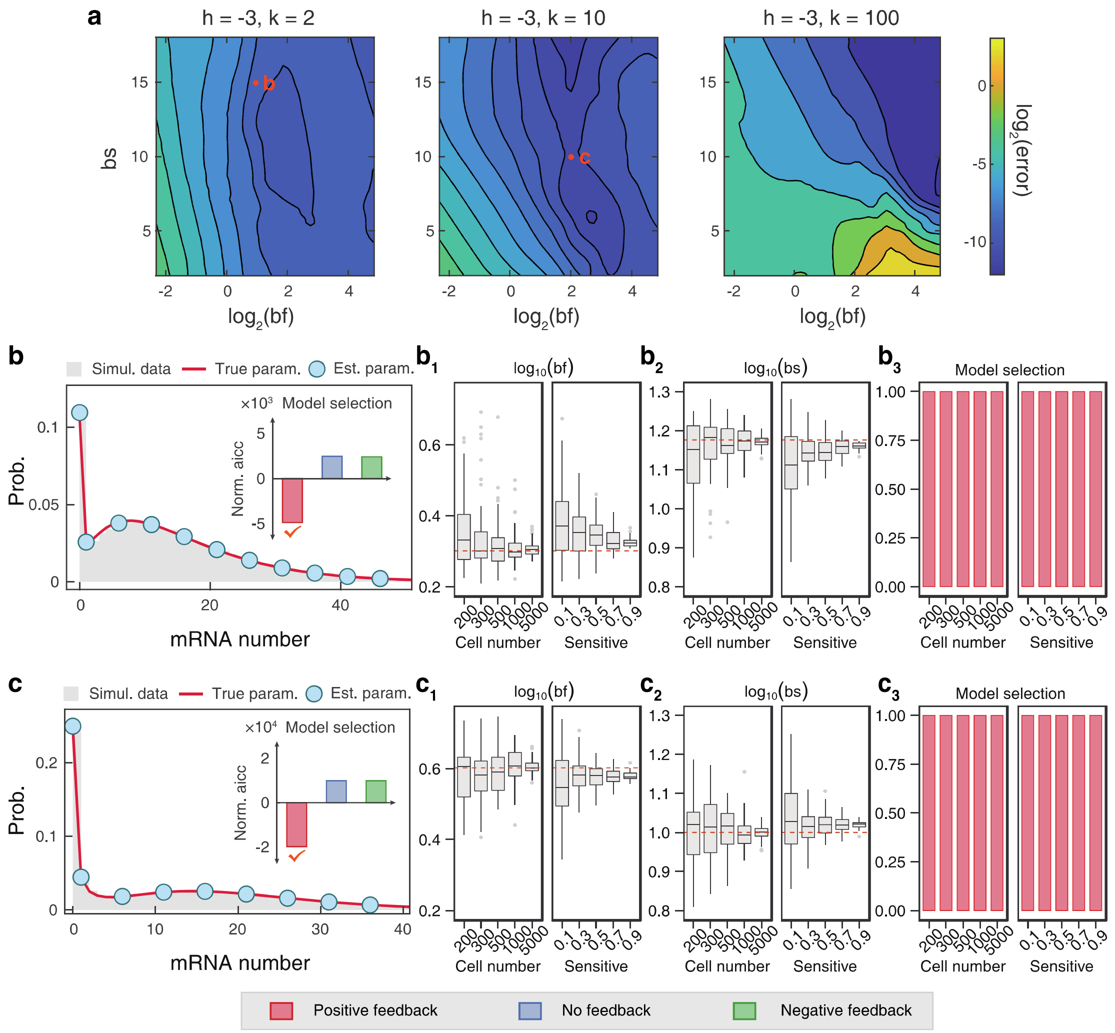

Supplementary Figure S5. Precision and robustness of inferred results in the case of positive feedback. (a) Contours illustrate the errors (color bars) of inferred burst kinetics for simulated gene-expression data generated by setting different equilibrium binding constants $k = 2,\ 10,\ 100$ (from left to right) and $h = -3$. The orange dots represent burst frequencies and sizes for the examples shown in (b) and (c). **(b)** mRNA distributions for fixed $a = 2$, $b = 15$, where the gray histograms are for simulated data, red solid line for the distribution generated using true kinetic parameters and blue points for the distribution generated using estimated kinetic parameters; Inset: analysis of model selection based on the AICc that is normalized for visualization convenience, where three colored bars represent the results of model selection for three cases of different feedbacks and the check mark represents the final selected model. **(b_1-b_3)** Boxplot of inferred burst frequencies, burst sizes and feedback models as functions of different cell numbers and random deletions (sensitive), where the red dashed line corresponds to the true parameter. **(c and c_1-c_3)** Analysis of robustness inference similar to b and b_1-b_3 for fixed $a = 4$, $b = 10$.

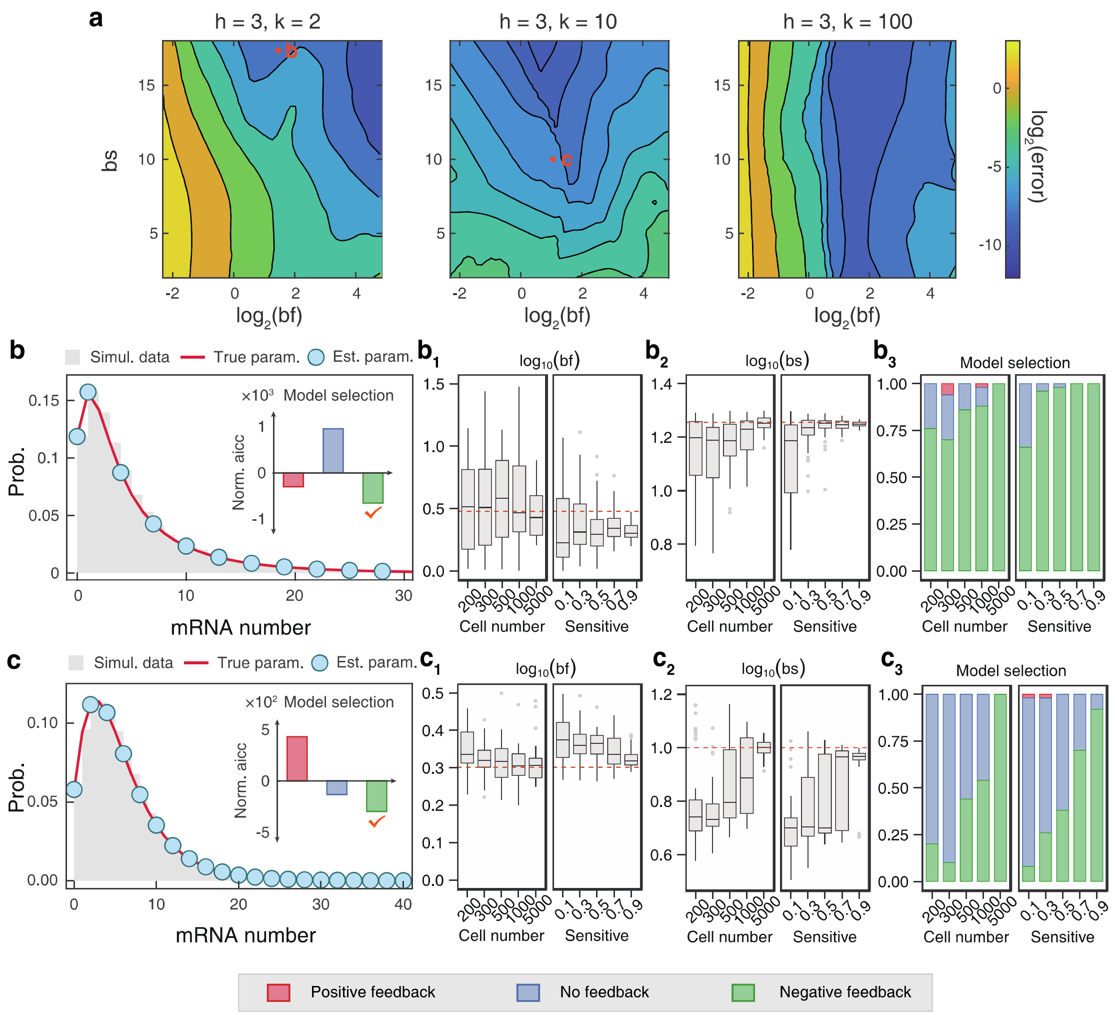

Supplementary Figure S6. Precision and robustness of inferred results in the case of negative feedback. (a) Contours illustrate the errors (color bars) of inferred burst kinetics for simulated gene-expression data generated by setting different equilibrium binding constants $k = 2,\ 10,\ 100$ (from left to right) and $h = 3$. The orange dots represent burst frequencies and sizes for the examples shown in (b) and (c). **(b)** mRNA distributions for fixed $a = 3$, $b = 18$, where the gray histograms are for simulated data, red solid line for the distribution generated using true kinetic parameters and blue points for the distribution generated using estimated kinetic parameters; Inset: analysis of model selection based on the AICc that is normalized for visualization convenience, where three colored bars represent the results of model selection for three cases of different feedback and the check mark represents the final selected model. **(b_1-b_3)** Boxplot of inferred burst frequencies, burst sizes and feedback models as functions of different cell numbers and random deletions (sensitive), where the red dashed line corresponds to the true parameter. **(c and c_1-c_3)** Analysis of robustness inference similar to b and b_1-b_3 for fixed $a = 2$, $b = 10$.

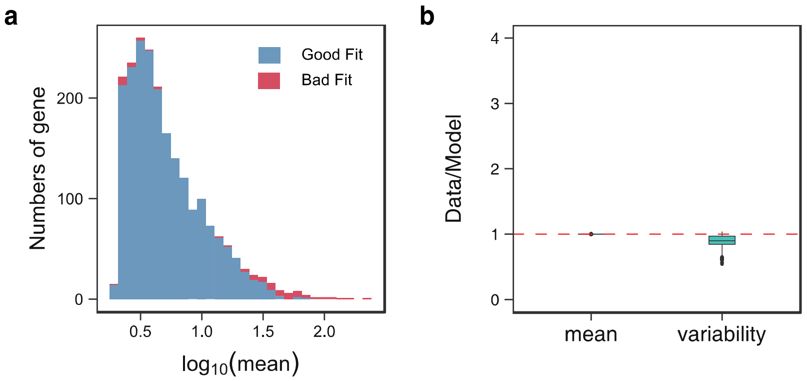

Supplementary Figure S7. Results for performing goodness-of-fit test analysis. **(a)** Demonstration of Goodness-fit test for all inferred genes, where the histogram shows the gene numbers with good-fit (blue color) and bad-fit (red color) vs the mean expression levels. **(b)** Comparison of the mean expression level and the variability (CV^2) between the scRNA-seq dataset and the hierarchical model, where the red dashed line represents those results for the model and the data are completely consistent.

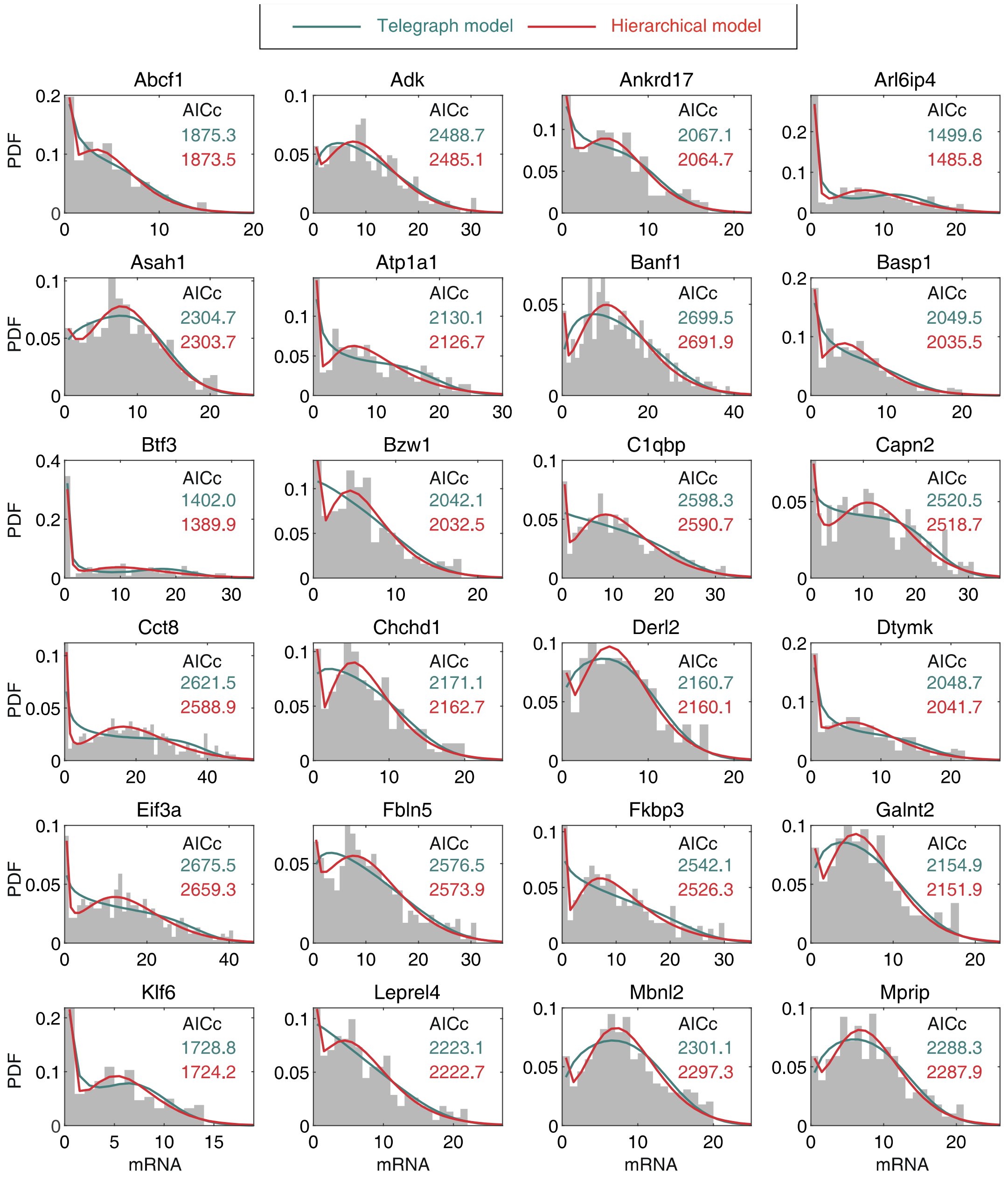

Supplementary Figure S8. Comparison of fitting and model selection between hierarchical and telegraph models of bimodal distribution. Bimodal examples for comparison of the inferred distributions between our hierarchical model (orange line) and the telegraph model (green line), where the gray histograms represent the distributions of mRNA counts. Corrected Akaike information criterion (AICc) is calculated for model selection.

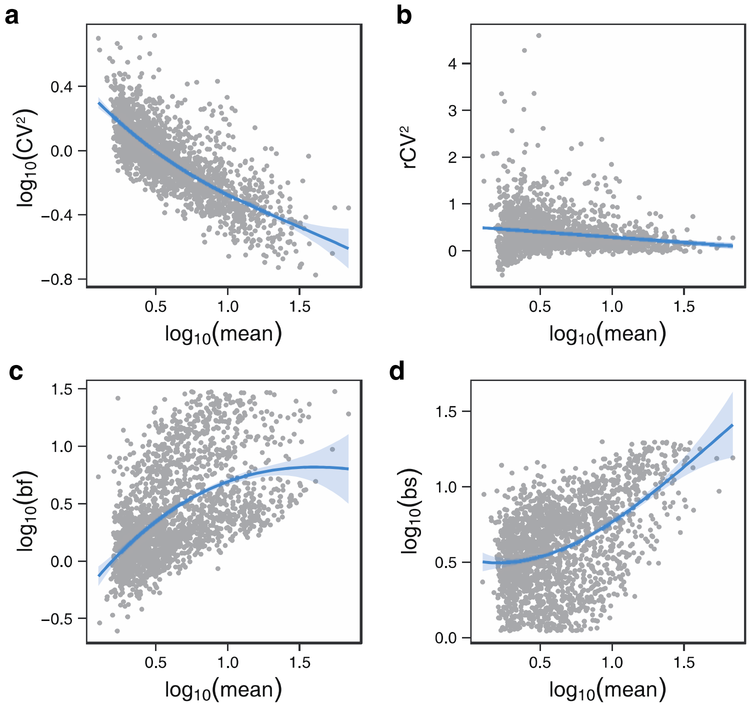

Supplementary Figure S9. Genome-wide inference of the relationships between kinetic parameters and mean expression levels. (a) Scatter plot of mean gene-expression levels against expression variability (CV^2) for all genes. **(b)** Scatter plot of mean gene-expression levels against rCV^2 for all genes. **(c)** Scatter plot of mean gene-expression levels against burst frequencies for all genes. **(d)** Scatter plot of mean gene-expression levels against burst sizes for all genes. In a-d, the solid lines represent the fitting curves with a cubic spline and the shading represents 95% confidence interval.

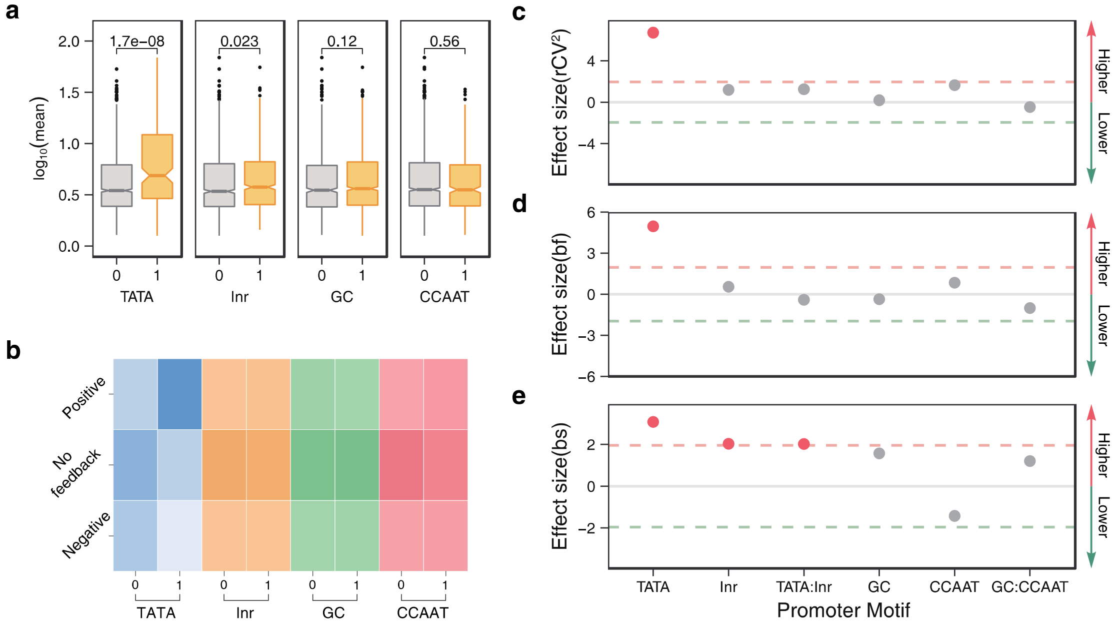

Supplementary Figure S10. Correlations among promoter motifs, feedbacks and burst kinetics. (a) Boxplots of the mean expression levels of genes either with (1) or without (0) promoter motifs, where p-value represents the result of Wilcoxon test. **(b)** Correlations between promoter motifs and feedback regulations, where the shades of the color indicate the frequencies in the contingency table, which are normalized by x axis. **(c-e)** Results for the regressions of variability (rCV^2**,** c), burst frequencies (d) and burst sizes (e) by using promoter motif features, where each point shows the t-value in a multivariate linear regression model, which is used to judge whether to reject the null hypothesis that the feature has no connection with the dependent variable. Color: Significantly higher (red point), significantly lower (green point) and no significant (gray point) effects.

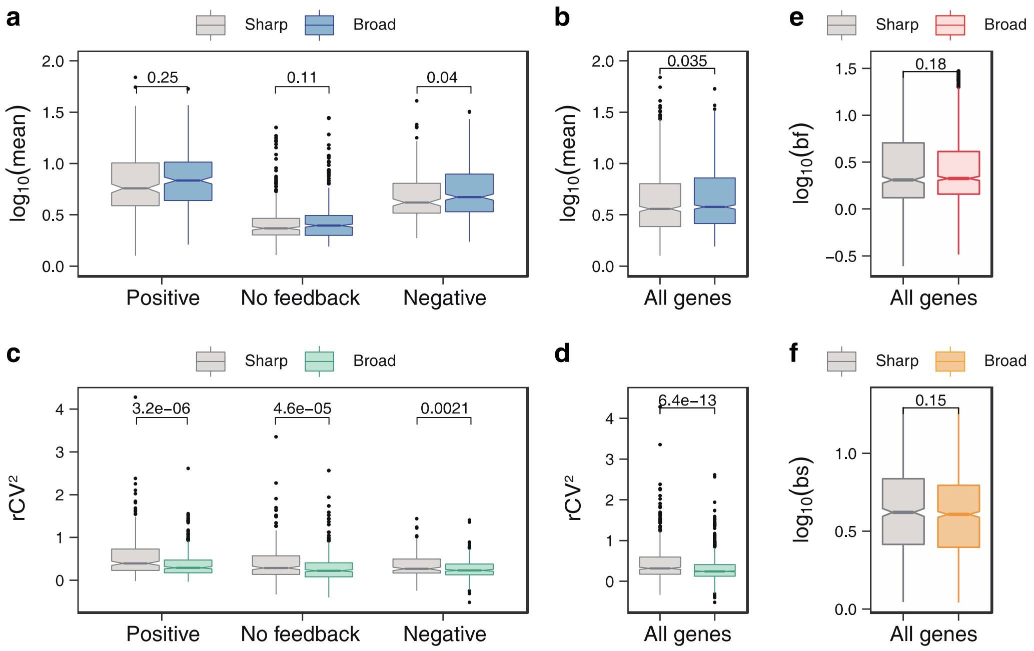

Supplementary Figure S11. Correlations among TSS distributions, feedbacks and burst kinetics. (a,b) Boxplots of mean expression level of genes either with sharp TSS distribution or with broad TSS distribution in three cases of different feedbacks (a) and for all genes (b), where the p-values represent the results of Wilcoxon test. **(c,d)** Boxplots of variability of genes either with sharp TSS distribution or with broad TSS distribution in three cases of different feedbacks (c) and for all genes (d). **(e,f)** Boxplots of burst frequency (e) and burst size (f) of genes either with sharp TSS distribution or with broad TSS distribution for all genes.

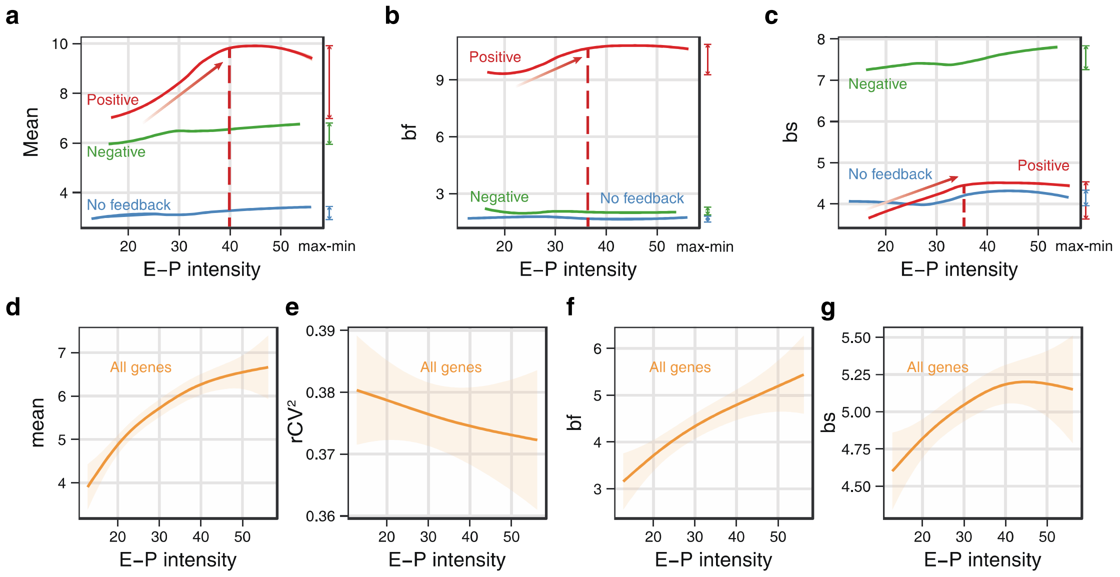

Supplementary Figure S12. Synergic effects of enhancer-promoter interaction and feedback regulation on burst kinetics. (a-c) Correlations between enhancer-promoter (E-P) interaction intensity and mean gene-expression levels (a), burst frequencies (b) or burst sizes (c) in three cases of different feedbacks, where the dashed line represents the peak value in case of positive feedback and the line segment on the right-hand side of the picture represents the maximum minus the minimum, that is, the amplitude of affecting kinetic parameters (mean levels, burst frequencies and burst sizes). Color: positive feedback (red line), non-feedback (blue line) and negative feedback (green line). **(d-g)** Correlations between E-P interaction intensity and mean gene-expression levels (d), variability ($r\mathrm{CV}^2$**,** e), burst frequencies (f) or burst sizes (g) for all genes.

3. Supplementary Table

Supplementary Table S1: The predicted feedback loop of genes that verified by publications.

Gene name	Feedback Type	PMID (or DOI)	Gene name	Feedback Type	PMID (or DOI)
Acta1	Positive	32087752	Cdkn1a	Positive	20160708
Acta2	Positive	26559755	Cebpd	Positive	25591788
Actn1	Positive	16736472	Clic1	Positive	32905514
Adamts5	Positive	18328163	Clic4	Positive	31879279
Adipor1	Positive	33359123	Cnbp	Positive	28168305
Aebp1	Positive	32565808	Cnn2	Positive	24464300
Ahnak	Positive	21940993	Col6a3	Positive	33214660
Akt1	Positive	26235620	Commd1	Positive	26586569
Aldh18a1	Positive	32075946	Ctgf	Positive	23259531
Aldoa	Positive	32530543	Ctsl	Positive	33919392
Angptl2	Positive	33747748	Cxcl5	Positive	35116536
Ankrd11	Positive	20452957	Ddah1	Positive	23717555
Anxa2	Positive	33712571	Dhx9	Positive	35590370
Aprt	Positive	16105024	Ecm1	Positive	27075243
Arpc4	Positive	34836783	Eif3i	Positive	25147179
Atf5	Positive	29137451	Ensa	Positive	21164013
Basp1	Positive	33042262	Ephx1	Positive	30293161
Bdnf	Positive	25171395	Fasn	Positive	21643005
Bgn	Positive	33117706	Fhl2	Positive	16343438
Brd2	Positive	21068722	Fstl1	Positive	18519848
Bsg	Positive	10.1101/2021.11.29.470317	Fxyd5	Positive	31746425
			Fzd7	Positive	35942683
Btg2	Positive	11989967	Gng10	Positive	34429144
Cald1	Positive	26926107	Gng11	Positive	26926107
Cap1	Positive	34099549	Golm1	Positive	35805075
Ccdc104	Positive	23303910	Grb10	Positive	15901248
Cck	Positive	12829630	Hbegf	Positive	31033049
Ccl2	Positive	27888616	Hbp1	Positive	28348080
Ccl7	Positive	35715847	Hdgf	Positive	26296979
Cct2	Positive	33268369	Hmga1	Positive	31814893
Cd81	Positive	10.1101/2022.02.23.481674	Hnrnpc	Positive	35661832
			Hnrnpm	Positive	35158098
Cdc37	Positive	12435747	Htra1	Positive	22949504
Cdc42se1	Positive	30754684	Il1rl1	Positive	35578178
Cdk1	Positive	22726437	Klf6	Positive	33734616
Cdk4	Positive	10235368	Ldha	Positive	35359405
Cdk5	Positive	11050161	Lox	Positive	21643005

(Continued to Supplementary Table S1)

Gene name	Feedback Type	PMID (or DOI)	Gene name	Feedback Type	PMID (or DOI)
Ltbr	Positive	29769272	Wnk1	Positive	35691270
Malat1	Positive	26735578	Ythdf2	Positive	34160549
Meg3	Positive	33602903	Abi1	Negative	11208021
Mmp14	Positive	31298339	Adam9	Negative	34671207
Ncl	Positive	25938538	Alas1	Negative	29364890
Neat1	Positive	33253679	Anxa1	Negative	27105503
Nisch	Positive	35108640	Atf4	Negative	27629041
Pak2	Positive	12226077	Bin1	Negative	30733337
Pdrg1	Positive	34812552	Bmp1	Negative	25701650
Pgk1	Positive	30537744	Calm1	Negative	29657261
Plk2	Positive	29448085	Camk2g	Negative	16002660
Postn	Positive	10.1161/CIRCRESAHA.120.316943	Ccm2	Negative	29364115
			Chd4	Negative	25453762
			Chpf	Negative	33809195
Prdx6	Positive	31007045	Ckap4	Negative	27322059
Psmd1	Positive	10.1182/blood-2019-126963	Col4a2	Negative	35532293
			Dad1	Negative	31123095
Psmd3	Positive	10.1182/blood-2019-126963	Ddr2	Negative	22832484
			Ddx24	Negative	24204270
Ptpn11	Positive	30355677	Dhrs3	Negative	28207193
Rbfox2	Positive	31241461	Dnajc3	Negative	33210431
Rock2	Positive	27724862	Drap1	Negative	12471260
Rpl23	Positive	28539603	Eif3h	Negative	29136179
Rraga	Positive	35739524	Erp29	Negative	21419175
S100a10	Positive	30675195	Fabp5	Negative	31883840
Sdpr	Positive	30879950	Gas5	Negative	35706414
Serpine2	Positive	33760135	Gba	Negative	33617695
Sh3bgrl3	Positive	33839406	Gpc1	Negative	31355137
Son	Positive	27293512	Herc2	Negative	31665549
Sox4	Positive	30906628	Hnrnpa0	Negative	35879279
Sparc	Positive	18542844	Iqgap1	Negative	22328503
Tagln2	Positive	27402267	Kras	Negative	24755884
Thbs1	Positive	32853092	Lamp1	Negative	29644770
Timp1	Positive	35961511	Lats2	Negative	27006470
Tspo	Positive	33066460	Loxl1	Negative	29164236
Uba2	Positive	35661832	Ly6e	Negative	31684192
Unc5b	Positive	32141538	Map2k2	Negative	29023632
Uqcr10	Positive	34860557	Nedd4	Negative	31787758
Wisp2	Positive	30651114	Nolc1	Negative	24154670

(Continued to Supplementary Table S1)

Gene name	Feedback Type	PMID (or DOI)
Npm1	Negative	23874639
Nrp2	Negative	29130509
Pgd	Negative	33241112
Prrc1	Negative	34295164
Rab7	Negative	29296513
Rab8a	Negative	22219378
Rac1	Negative	27136688
Rb1cc1	Negative	33340270
Rbm39	Negative	10.1101/2022.08.30.505862
Rdx	Negative	16651542
Rhoa	Negative	27136688
Rpl22	Negative	23990801
Rps3	Negative	20217897
Sar1a	Negative	10.1101/2022.07.21.501000

Gene name	Feedback Type	PMID (or DOI)
Smarca4	Negative	30297712
Srsf2	Negative	25818199
Ssrp1	Negative	26755331
Stat3	Negative	24973454
Tardbp	Negative	33137311
Thoc7	Negative	10.1101/2021.09.07.459221
Tmed9	Negative	35805075
Tnk2	Negative	26677978
Trim3	Negative	30542119
Trip12	Negative	22497224
Trip6	Negative	32104300
Tspan31	Negative	24565034
Ubxn4	Negative	34021047
Usp14	Negative	30021169

4. Reference

1. Sarkar, A. and Stephens, M. (2021) Separating measurement and expression models clarifies confusion in single-cell RNA sequencing analysis. *Nat. Genet.*, **53**, 770-777.

2. Raharinirina, N.A., Peppert, F., von Kleist, M., Schütte, C. and Sunkara, V. (2021) Inferring gene regulatory networks from single-cell RNA-seq temporal snapshot data requires higher-order moments. *Patterns*, **2**, 100332.

3. Liu, Y., Beyer, A. and Aebersold, R. (2016) On the dependency of cellular protein levels on mRNA abundance. *Cell*, **165**, 535-550.

4. To, T.-L. and Maheshri, N. (2010) Noise can induce bimodality in positive transcriptional feedback loops without bistability. *Science*, **327**, 1142-1145.

5. Gupta, A., Martin-Rufino, J.D., Jones, T.R., Subramanian, V., Qiu, X., Grody, E.I., Bloemendal, A., Weng, C., Niu, S.-Y. and Min, K.H. (2022) Inferring gene regulation from stochastic transcriptional variation across single cells at steady state. *Proc. Natl. Acad. Sci. U. S. A.*, **119**, e2207392119.

6. Shahrezaei, V. and Swain, P.S. (2008) Analytical distributions for stochastic gene expression. *Proc. Natl. Acad. Sci. U. S. A.*, **105**, 17256-17261.

7. Friedman, N., Cai, L. and Xie, X.S. (2006) Linking stochastic dynamics to population distribution: an analytical framework of gene expression. *Phys. Rev. Lett.*, **97**, 168302.

8. Peccoud, J. and Ycart, B. (1995) Markovian modeling of gene-product synthesis. *Theor. Popul. Biol.*, **48**, 222-234.

9. Kim, J.K. and Marioni, J.C. (2013) Inferring the kinetics of stochastic gene expression from single-cell RNA-sequencing data. *Genome Biol.*, **14**, R7.

10. Hildebrand, B.F. (1987) *Introduction to numerical analysis*. 2nd ed. Dover, New York.
